# Doxorubicin- and Selenium-Incorporated Mesoporous Silica Nanoparticles as a Combination Therapy for Osteosarcoma

**DOI:** 10.1021/acsanm.4c04294

**Published:** 2024-11-01

**Authors:** Lei He, Zahra Javid Anbardan, Pamela Habibovic, Sabine van Rijt

**Affiliations:** Department of Instructive Biomaterials Engineering, MERLN Institute for Technology Inspired Regenerative Medicine, Maastricht University, P.O. Box 616, 6200 MD Maastricht, The Netherlands

**Keywords:** combination therapy, selenium, doxorubicin, mesoporous silica nanoparticles, hyaluronic acid, pH-responsivity

## Abstract

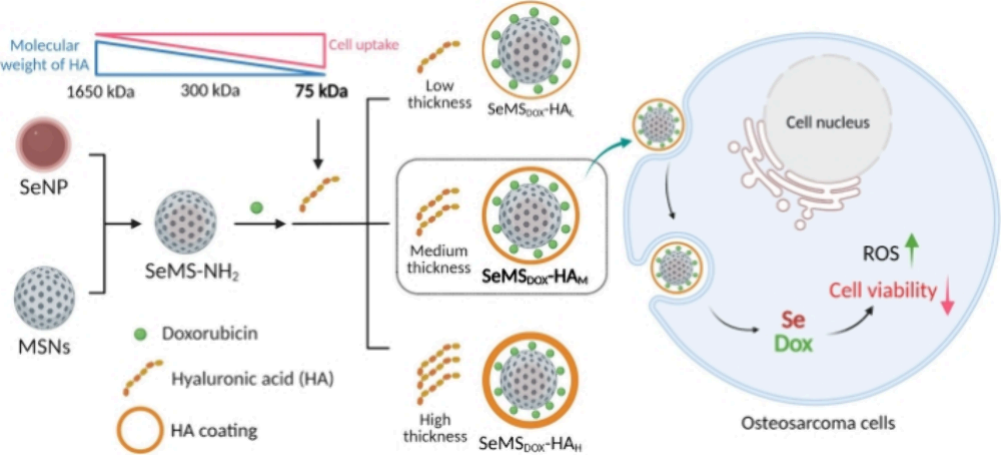

Doxorubicin (Dox) is a promising anticancer chemotherapeutic, which has been widely investigated in osteosarcoma (OS) treatment. However, there are several disadvantages regarding its clinical use. Specifically, Dox has low specificity toward cancer cells, which can lead to serious side effects. In addition, cancer cells can develop resistance toward Dox, reducing its therapeutic efficiency. Combination therapy (CT) facilitated by nanoparticle delivery systems is a promising strategy to overcome these drawbacks. In this study, we investigated the effectiveness of Dox and selenium (Se) CT using mesoporous silica nanoparticles (MSN) coated with hyaluronic acid (HA) as drug carriers. We hypothesized that combining Se as a second agent can increase Dox anti-OS effectiveness and that MSN can be used to facilitate dual drug delivery. In our system, HA was used as a gatekeeper to control the intracellular release of Se/Dox by means of its pH-responsive degradation. CT therapy using MSNs coated with HA led to a higher OS inhibitory efficiency in vitro compared to MSNs carrying either Se or Dox alone. This study demonstrates that using MSNs for the dual delivery of Se and Dox is a promising method for OS therapy.

## 1 Introduction

Malignant osteosarcoma (OS) is the most common primary bone cancer, which is predominantly diagnosed in adolescents and the elderly.^1^ OS not only reduces the patients’ life expectancy but also causes local pain, swelling, and limited range of motion.^2^ The standard treatment for OS comprises localized surgery and neoadjuvant chemo-radiotherapy, after which patients have a 5-year survival rate of 60–65%.^3^ Doxorubicin (Dox) is one of the most commonly used chemotherapeutic agents in OS (neo)adjuvant therapy in clinical practice.^4^ Dox has been acknowledged to target the cell nucleus and interfere with the cell replication process by suppressing the topoisomerase IIα-mediated pathway for DNA replication.^5^ However, Dox lacks specificity toward tumor cells, leading to systemic toxicity and serious side effects, such as cardiotoxicity.^6^ Additionally, the potential development of drug resistance in OS cells hampers the development of efficient OS treatments.^7^ For example, continuous Dox exposure induced tumor cells to upregulate topoisomerase IIα expression, thereby preventing nuclear DNA damage and apoptosis caused by Dox.^8^ In clinical practice, this means that OS cells in the lesion cannot be completely eliminated during treatment, potentially resulting in recurrence and metastasis of OS, which decreases the 5-year survival rate to less than 30%.^9^ The development of innovative strategies for OS therapy that address these issues is therefore needed.

Combination therapy (CT) is a promising strategy to enhance the therapeutic efficiency of individual chemotherapeutic agents. CT uses multiple treatment modalities with either similar or different mechanisms of action, with the goal of improving treatment outcomes by decreasing the therapeutic needed dose of individual drugs while amplifying the therapeutic effects. Several chemotherapeutic drugs (e.g., cisplatin, methotrexate, and sorafenib) have been employed in (neo)adjuvant regimens alongside Dox as CT, leading to better outcomes.^10−13^ Selenium (Se), a trace micronutrient in the human body, is considered a promising anti-OS compound due to its redox properties.^14^ Cell apoptosis is associated with oxidative stress caused by excessive ROS accumulation.^15^ By reacting with glutathione (GSH) and NADPH, Se can generate super anion radicals (·O_2_^–^), inducing intracellular oxidative stress, as reported by Zhao et al.^16^ Recently, a few studies demonstrated that Se enables to synergistically enhance the antitumor effect of Dox and overcome Dox resistance in several types of cancers.^17,18^ However, the controlled intracellular codelivery of Se/Dox is key to ensure their therapeutic effectiveness, since low cell uptake efficacy can induce extracellular accumulation, resulting in toxic effects on healthy cells.

Nanoparticle-based drug delivery vehicles offer a promising approach for codelivery of chemotherapeutics to cancer cells, thereby improving the therapeutic effectiveness of CT.^19^ Moreover, nanoparticles can be used to protect agents from degradation and control their release at the target site.^20^ In this regard, mesoporous silica nanoparticles (MSN) show great properties for CT, which include biocompatibility, biostability, large mesopores volume, and tunable (surface) structure.^21^ These features enable efficient loading of dual-therapeutic agents and facile surface functionalization to allow controlled drug delivery. For example, MSNs can be coated with stimuli-responsive polymers that cover the mesopores, acting as “gatekeepers” that only release drugs when exposed to certain stimuli. However, the application of MSNs for the controlled codelivery of Se and Dox in OS therapy remains underexplored, especially regarding the synergistic effects of Se and Dox compared to their individual monotherapies.

**Figure 1:**
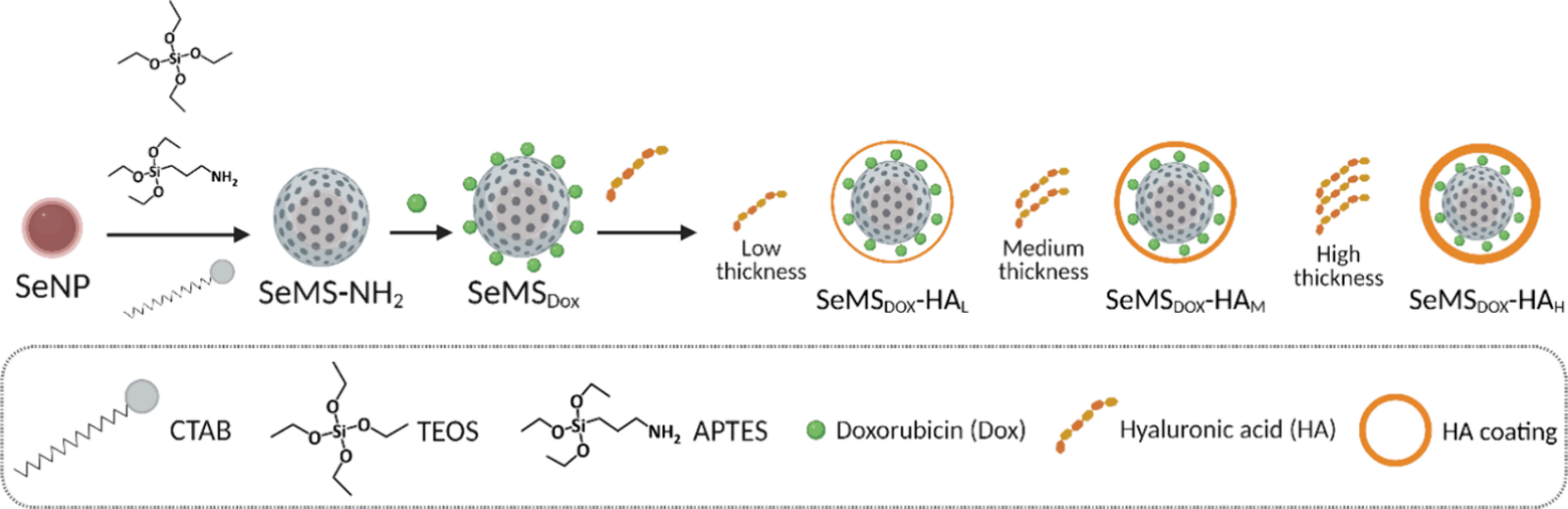
Schematic overview of the synthesis and optimization of SeMS_Dox_-HA, highlighting the hyaluronic acid (HA) coating thickness.

In this study, we investigated whether MSN can be used as a suitable drug delivery carrier for dual encapsulation of Se/Dox for CT of OS. Se nanoparticles (SeNPs), the zerovalent form of Se, was selected as the Se source as it has been shown to exhibit greater ROS-generating efficiency and lower toxicity compared to ionic/organic Se.^22^ The SeNPs were surface coated with mesoporous silica (creating SeMS) to allow Dox loading in the mesopores (SeMS_Dox_). The SeMS_Dox_ was further surface modified with hyaluronic acid (HA), a natural unbranched polysaccharide, as a gatekeeper to enable controlled Dox/SeNP delivery due to pH-sensitive hydrolysis of glycosidic bonds.^23−25^ In addition, HA can strongly target the CD44 receptor, which is highly overexpressed on the membranes of OS cells, making HA a widely employed surface ligand for cancer-targeting nanoparticles.^26^ The nanoparticles were first optimized in terms of Dox concentration, HA molecular weight (MW), and HA coating thickness to establish important design rules for their formulation (Figure 1). Next, the importance of these parameters as well as the mono- versus dual therapy was investigated in vitro. Here we show that HA-coated SeMS nanoparticles loaded with Dox, represent a promising CT for OS.

## 2 Materials and Methods

### 2.1 Chemicals

Tetraethyl orthosilicate (TEOS), 3-mercaptopropyl triethylsilane (MPTES), triethanolamine (TEA), 3-aminopropyl triethoxysilane (APTES), sodium selenite (Na_2_SeO_3_), ammonium nitrate (NH_4_NO_3_), ATTO-647 maleimide fluorophore, l-ascorbic acid (*V*_c_), cetyltrimethylammonium chloride (CTAC), cetyltrimethylammonium bromide (CTAB), ammonium fluoride (NH_4_F), ammonium nitrate (NH_4_NO_3_), and hydrochloric acid (HCl, 37%) for nanoparticle synthesis were purchased from Sigma-Aldrich GmbH (Germany). Absolute ethanol was purchased from VWR (US). Doxorubicin (Dox) from Sigma-Aldrich GmbH (Germany), hyaluronic acid (HA) sodium salt (50–100, 200–400, and 1500–1800 kDa) from Glentham Life Science (UK), 1-ethyl-3-(3-(dimethylamino)propyl) carbodiimide hydrochloride (EDC) from Thermo Fisher Scientific (US), and *N*-hydroxysulfosuccinimide (Sulfo-NHS) from abcr GmbH (Germany) were used for nanoparticle modification. Sodium cacodylate trihydrate ((CH_3_)_2_AsO_2_Na·3H_2_O), glutathione (GSH), and reduced nicotinamide adenine dinucleotide phosphate (NADPH), and NHS-FITC, used for nanoparticle characterization, were obtained from Sigma-Aldrich GmbH (Germany). Saos-2 and U2-OS cells were purchased from ATCC (US). Human mesenchymal stem cells (hMSCs) were purchased from Lonza (Switzerland). CellTiter 96 AQ_ueous_ nonradioactive cell proliferation (dimethylthiazol-carboxymethoxyphenyl-sulfophenyl-tetrazolium; MTS) assay from Promega (US) was used for the cell viability test. Paraformaldehyde was purchased from Sigma-Aldrich GmbH (Germany) for cell fixation. DAPI (4′, 6-diamidino-2-phenylindole) was bought from Thermo Fisher Scientific (US). The DCFDA/H_2_DCFDA assay kit was purchased from Abcam (UK) for ROS measurement.

### 2.2 Synthesis and Characterization of MSNs Coated with Different Molecular Weights (MW) of HA (MSNs-HA_75_, MSNs-HA_300_, and MSNs-HA_1650_)

MSNs with thiol/amine functionalization were synthesized as per our previously published method (shown in Supporting Information Scheme S1).^27^ For the conjugation of HA to the MSN surface, we employed an EDC reaction to link the −NH_2_ groups on MSN with the −COOH groups of HA.^28^ Briefly, 2 mg of HA of varying MW (HA_75_, HA_300_, and HA_1650_) was dissolved in water (pH 7.4), under mild stirring. This was followed by the addition of EDC (9.6 mg) to activate the −COOH groups in HA. After 45 min, Sulfo-NHS (10.9 mg) was introduced to the mixture and stirring was continued for an additional 45 min. The pH was then adjusted to 8.5, and MSNs (10 mg) were added. Stirring was maintained overnight to form MSNs-HA_75_, MSNs-HA_300_, and MSNs-HA_1650_ (Table 1). These nanoparticles were then collected, washed three times with ethanol, and stored at −20 °C under dry conditions.

**Table 1:** Summary of All Groups of Synthesized Nanoparticles

sample code	description	HA molecular weight (kDa)	SeMS: HA ratios (w/w)
MSNs-HA_75_	MSNs coated with low MW HA	50–100	1:0.2
MSNs-HA_300_	MSNs coated with medium MW HA	200–400	1:0.2
MSNs-HA_1650_	MSNs coated with high MW HA	1500–1800	1:0.2
SeMS-HA_L_	SeMS coated with low ratio of HA (low MW)	50–100	1:0.05
SeMS-HA_M_	SeMS coated with middle ratio of HA (low MW)	50–100	1:0.1
SeMS-HA_H_	SeMS coated with high ratio of HA (low MW)	50–100	1:0.2
SeMS_Dox_-HA_L_	SeMS loaded with Dox and coated with low ratio of HA	50–100	1:0.05
SeMS_Dox_-HA_M_	SeMS loaded with Dox and coated with middle ratio of HA	50–100	1:0.1
SeMS_Dox_-HA_H_	SeMS loaded with Dox and coated with high ratio of HA	50–100	1:0.2
MS_Dox_-HA_M_	MSNs loaded with Dox and coated with middle ratio of HA	50–100	1:0.1
MS_Dox_-HA_H_	MSNs loaded with Dox and coated with high ratio of HA	50–100	1:0.2

Transmission electron microscopy (TEM; FEI electron microscope, US) and dynamic light scattering (DLS; Malvern Zetasizer Nano, Panalytical, UK) were used to characterize the morphology and surface charge (zeta potentials, ζ) of MSNs-HA. The size (diameter) of nanoparticles was measured using TEM images and ImageJ. In addition, Fourier transform-infrared spectroscopy (FTIR; Nicolet iS50 spectroscope) was utilized for functional groups analysis of SeMS-HA, recording spectra from 4000 to 400 cm^–1^. To fully evaluate the HA coating, an NHS-FITC fluorophore labeling method was used to measure the −NH_2_ groups in the modified MSNs. Briefly, 20 μL of NHS-FITC (5 mg/mL) was added to 1 mg of nanoparticles in 1 mL of ethanol for continuous stirring overnight in the dark. The nanoparticles were washed three times with absolute ethanol and subsequently resuspended in 96-well plates for fluorescence analysis by a CLARIOstar spectrophotometer equipped with MARS data analysis software (BMG LABTECH, Germany).

### 2.3 ATTO-647 Labeled MSNs-HA uptake in OS Cells

The presence of −SH groups in MSNs allows for the labeling with ATTO-647 maleimide fluorophore. This labeling process was performed prior to the HA coating of MSN. Specifically, MSNs (1 mg) were resuspended in absolute ethanol at room temperature, under mild stirring. Then, 5 μL of ATTO-647 maleimide fluorophore solution (5 mg/mL) was added, and the mixture was stirred continuously overnight in the dark. The ATTO-647 labeled MSNs were then washed three times with ethanol and water, followed by the HA coating as previously described. The fluorescence of ATTO-647 labeled MSNs-HA, including MSNs-HA_75_, MSNs-HA_300_, and MSNs-HA_1650_, was detected using a BIO-RAD microplate reader 550.

Next, all variants of ATTO-647 labeled MSNs-HA were sterilized using 70% ethanol and subsequently washed with DMEM medium. OS cells, including Saos-2 and U2OS cells, cultured in flasks were harvested and seeded in 12-well plates at a density of 1 × 10^5^/well in 1 mL. After 24 h incubation, the medium was replaced with fresh medium containing dye-labeled MSNs-HA at a concentration of 200 μg/mL. Both Saos-2 and U2OS cells were cultured with MSNs-HA for 24 and 72 h. Then, the medium with nanoparticles was discarded, and cells were washed twice with PBS. The cells were then harvested, washed, and resuspended in PBS before being transferred into flow cytometer (FACS) tubes. The fluorescence was quantified using a flow cytometer (AZM BD FACS Canto II, US).

### 2.4 Synthesis of SeMS, SeMS-HA_,_ SeMS_Dox_-HA, and MS_Dox_-HA

A one-pot method published previously was employed for SeMS synthesis (shown in Supporting Information Scheme S2).^29^ SeMS were surface-modified by HA through an EDC reaction, similar to that of MSNs. To optimize the HA surface modification of SeMS or MSNs, the SeMS (or MSN): HA ratios (w/w) of 1:0.05, 1:0.1, and 1:0.2, were used. Briefly, HA at quantities of 0.5, 1, and 2 mg was first activated by EDC (2.4, 4.8, and 9.6 mg) in water (pH 7.4) with continuous stirring. This was followed by adding Sulfo-NHS (2.7, 5.5, and 10.9 mg) to the mixture. After adjusting the pH of the mixture to 8.5, SeMS (10 mg) was introduced and stirred overnight. The resulting SeMS-HA was then collected, washed with water, and stored in the freezer. These samples were divided into three groups, labeled as SeMS-HA_L_, SeMS-HA_M_, and SeMS-HA_H_, corresponding to the SeMS/HA (w/w) ratios of 1:0.05, 1:0.1, and 1:0.2, respectively (Table 1).

To synthesize Dox-loaded SeMS coated with HA, we initially loaded Dox into the mesopores of MS prior to the HA coating process (Figure 1). Initially, 20 mg SeMS were redispersed in 3 mL of Milli-Q water, followed by the addition of 7 mL of 0.1 mg/mL Dox solution (Milli-Q water) and overnight stirring, to produce Dox-loaded SeMS (SeMS_Dox_). SeMS_Dox_ (20 mg) was followed by reacting with different amounts of activated HA_,_ as previously described. The nanoparticles were stirred overnight, collected, washed once with water, and stored in the freezer, labeled as SeMS_Dox_-HA. Three variants, SeMS_Dox_-HA_L_, _M_, and _H_, represented nanoparticles with three different amounts of HA coating as aforementioned (Table 1). Additionally, Dox-loaded MSNs coated with HA were prepared in the same way, serving as controls in the study. MS_Dox_-HA_M_ and _H_ represented the middle and high amounts of coating HA on MSNs (Table 1).

Characterization of the nanoparticles was performed by using TEM and DLS to assess the morphology and surface charge (zeta potentials, ζ). Additionally, FTIR was utilized for functional group analysis of SeMS-HA, recording spectra from 4000 to 400 cm^–1^. The aforementioned NHS-FITC fluorophore labeling method was used to measure the −NH_2_ groups in SeMS_Dox_-HA to prove the HA coating on SeMS_Dox_.

### 2.5 Analysis of Dox Loading and the pH-responsive Release Profile

The Dox content and loading efficacy in the nanoparticles were quantified using the following equations:
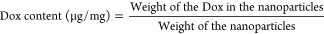
1
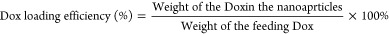
2The Cary Eclipse fluorescence spectrophotometer (Agilent Technologies; US) was employed to measure the fluorescence of Dox-loaded nanoparticles and the standard curves (λ_ex_ = 480 nm and λ_em_ = 560 nm).^30^ The weight of the Dox in the nanoparticles was calculated from the fluorescent intensity.

To evaluate the pH-responsive Dox release from nanoparticles, cacodylate buffer at pH 7.4 and 5.0 was used to simulate physiological and lysosomal conditions, correspondingly. Briefly, 3.5 mg of each nanoparticle were enclosed in Slide-A-Lyzer MINI Dialysis Devices (10K MWCO, Thermo Fisher; US) and immersed in 3.5 mL of respective pH buffer in BRAND macrocuvettes (Sigma-Aldrich GmbH; Germany). The cuvettes with nanoparticles were then sealed and placed in the fluorescence spectrophotometer maintained at 37 °C with mild stirring. These setups allowed only the small molecular Dox to pass through the membrane into the lower part of the cuvette. Real-time fluorescence measurements were conducted over a 72-h period to monitor Dox release.

### 2.6 Analysis of Se Loading and Release Profile

To evaluate Se release from SeMS_Dox_-HA, cacodylate buffer with pH 7.4, 5.0, and 7.4 with GSH (10 mM) and NADPH (1 mM) were used to mimic physiological and lysosomal conditions in OS cells. Se release was assessed by using a cumulative release model (**Scheme S2**). Briefly, 250 μg of SeMS_Dox_-HA_L_, SeMS_Dox_-HA_M_, and SeMS_Dox_-HA_H_ were enclosed in dialysis devices and immersed in neutral, acidic, and redox buffer in Eppendorf tubes and placed in a ThermoMixer with 1000 rpm stirring at 37 °C. One milliliter of the supernatant was collected from each Eppendorf tube after 2, 4, 8, 12, 24, 48, and 72 h, followed by the addition of 1 mL of fresh medium for further release. After that, the supernatant collected at each interval was filtered using 0.2 μm membranes and diluted 12 times in aqueous 1% HNO_3_ containing 20 ppb Sc. To investigate the total Se content in SeMS_Dox_-HA_L_, SeMS_Dox_-HA_M_, and SeMS_Dox_-HA_H_, 0.25 mg of nanoparticles were dissolved in 2 mL of aqua regia, followed by dilution 12 times with aqueous 1% HNO_3_. Se and silicon (Si) elemental content were measured via inductively coupled plasma mass spectrometry (ICP-MS; iCaP Q, Thermo Scientific, US), where 20 ppb scandium (Sc) was used as an internal standard. The Se content (Se mol %) in SeMS_Dox_-HA was quantified using the following equation^29^ :
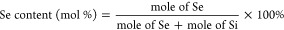
3

### 2.7 Cellular Internalization

The cellular uptake of SeMS_Dox_-HA_M_ and MS_Dox_-HA_M_ in OS cells was evaluated using fluorescent microscopy. MS_Dox_-HA_M_ containing–SH groups were labeled with ATTO-647-maleimide dye using the aforementioned method to localize the MSNs. The nanoparticles were sterilized with 70% ethanol and subsequently washed once with DMEM medium. Saos-2 and U2OS cells were harvested and cultured in 12-well plates at a density of 1 × 10^5^ cells/well (1 mL) for 24 h. Subsequently, a fresh medium containing SeMS-HA_M_ and MS_Dox_-HA_M_ (100 μg/mL) was introduced. Cells were then cultured with the nanoparticles for the additional 6 and 12 h. After incubation, cells were washed three times with PBS and fixed with 4% paraformaldehyde, followed by rinsing in PBS twice. Subsequently, 300 μL of DAPI solution (0.1 μg/mL in PBS) was introduced for 20 min incubation at RT. Then, the DAPI solution was discarded and cells were washed 2–3 times with PBS, followed by immersing cells in PBS for imaging using a fluorescence microscope (Eclipse Ti-E Nikon, Japan).

### 2.8 Cytotoxicity Assay on OS Cells

The cytotoxicity effects of nanoparticles on OS cells were evaluated by using the MTS assay. The nanoparticles, including SeMS-HA_M_, MS_Dox_-HA_M_, and SeMS_Dox_-HA_M_, were sterilized with 70% ethanol. Saos-2 and U2OS cells were cultured in 96-well plates at a density of 1 × 10^4^ cells/well (100 μL). After a 24 h incubation period, the medium in each well was replaced with fresh medium containing varying concentrations of nanoparticles (5, 25, 50, 75, 100, and 200 μg/mL). The cells were then incubated with nanoparticles for 24 and 72 h. Following the incubation, 20 μL of the MTS assay (MTS/PMS solution) was added to each well. The plates were incubated for an additional 2 h in the dark. The absorbance (O.D.) at 490 nm was measured using a CLARIOstar spectrophotometer. Cell viability (%) was calculated using the following equation:
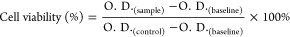
4where wells containing OS cells served as control, and cell-free SeMS-HA_M_, MS_Dox_-HA_M_, and SeMS_Dox_-HA_M_ served as the baseline.

### 2.9 Cytotoxicity of SeMS_Dox_-HA_M_ toward hMSCs

To investigate the cytotoxic effects of SeMS_Dox_-HA_M_, toward hMSCs, the MTS assay was performed. Briefly, hMSC cells were cultured in 96-well plates at a density of 1 × 10^4^ cells/well (100 μL) for 24 h. Subsequently, the cell medium was replaced by fresh medium containing SeMS_Dox_-HA_M_ at different concentrations (5, 25, 50, and 100 μg/mL) for 24 and 72 h incubation. After that, the MTS assay was used to measure the cell viability using the CLARIOstar spectrophotometer at 490 nm. Cell viability (%) was calculated using eq 4 mentioned previously.
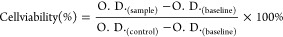
5Where wells with only hMSCs served as control and cell-free SeMS_Dox_-HA_M_ served as the baseline.

### 2.10 ROS Assay

The ROS assay was performed using the DCFH/DA kit as reported previously.^31^ Briefly, Saos-2 cells were cultured in 12-well plates at a density of 1 × 10^5^ cells/well in 1 mL medium overnight, followed by nanoparticle exposure using a medium containing 100 μg/mL of SeMS-HA_M_, MS_Dox_-HA_M_, or SeMS_Dox_-HA_M_. After 24 h of incubation, Saos-2 cells were washed twice with PBS and stained with DCFH/DA (10 μM; 1 mL) for 30 min in the dark. Subsequently, Saos-2 cells were washed, collected, and measured using a flow cytometer (AZM BD FACS Canto II, US).

### 2.11 Statistical Analysis

All results are shown as mean ± SD with at least *n* = 3. Statistical analysis was processed using GraphPad Prism 10 (US). Statistical significance (*: *p* < 0.005; **: *p* < 0.0001) was evaluated via the Student’s *t*-test (paired) and one/two-way analysis of variance (ANOVA) followed by Tukey’s multiple comparison test.

## 3 Results

### 3.1 Effect of HA Molecular Weight on Nanoparticle Cell Internalization

**Figure 2:**
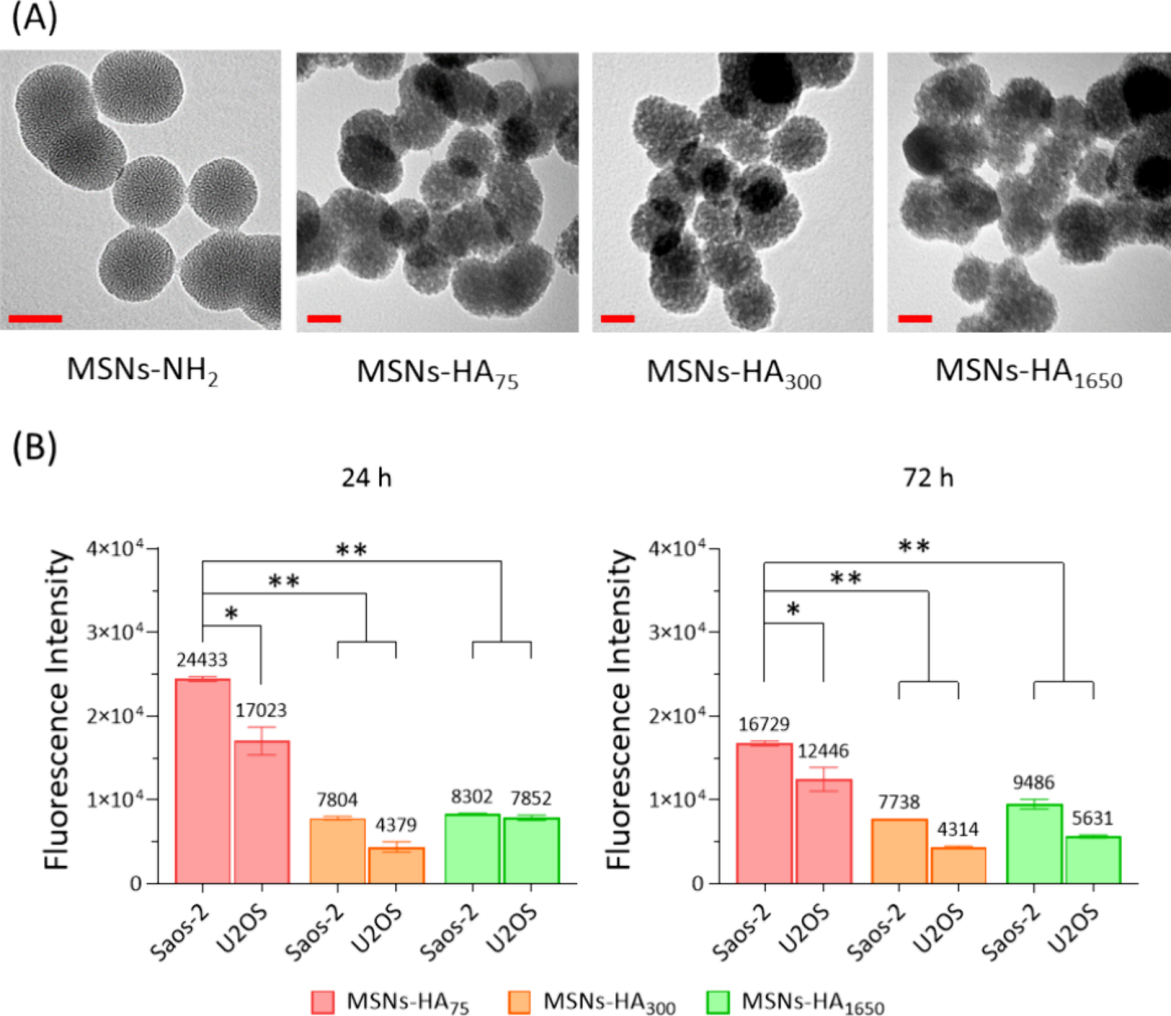
(A) TEM images of MSNs-NH_2_, MSNs-HA_75_, MSNs-HA_300_, and MSNs-HA_1650_; scale bar is 50 nm. (B) Fluorescence intensity histograms showing the cellular uptake of ATTO-647-maleimide labeled MSNs-HA in two different OS cell types after 24 and 72 h-exposure. All data are presented as mean ± standard deviation (*n* = 3). * presents statistical significance (*: *p* < 0.005; **: *p* < 0.0001).

The MW of HA can affect cell uptake efficiency.^32^ Therefore, we first aimed to investigate the effect of HA MW MSN coating on OS cell uptake in order to optimize nanoparticle therapeutic efficacy. We synthesized fluorescent dye-labeled MSNs that were coated with HA of three different MWs (75, 300, and 1650 kDa), and investigated their cell uptake efficacy in two different OS cell types: Saos-2 and U2OS. To develop the MSNs with thiols at their core and amines on their surface, an established co-condensation method was used.^27^ Subsequently, HA surface coating was achieved via an EDC coupling reaction of the free amines on the MSN surface with available carboxylic acid groups on HA. The synthesized MSN coated with HA and fluorescently labeled in their core were termed MSNs-HA_75_, MSNs-HA_300_, and MSNs-HA_1650._ Analysis using TEM revealed less visible and organized mesopores for MSN-HA compared to uncoated MSNs (Figure 2A). The MSNs-HA were larger than those of uncoated MSNs, with MSNs-HA_1650_ having a slightly larger size than MSNs-HA_75_ and MSNs-HA_300_ (Figure S1A). Moreover, their surface charge shifted from positive, due to the amine surface groups, to negative after coating with HA (Figure S1B), confirming that the MSNs were coated with HA. In addition, FTIR spectra revealed peaks at 1607, 1403, and 1152 cm^–1^ related to the C=O, C–O, and C–O–C stretching modes of HA^33^ (Figure S1C). We also performed a fluorescent labeling reaction to assay any remaining amine groups on the MSN surface. The absence of a fluorescent signal further proved that HA coating on MSNs was successful (Figure S1D). To visualize cellular uptake efficiency, MSNs were fluorescently labeled at their core with an ATTO-647 dye. Similar fluorescent intensities were observed for all three dye-labeled nanoparticles, confirming the successful conjugation of the ATTO-647 dye to the thiol-functionalized MSNs (Figure S1E).

Next, the nanoparticle uptake efficiency by OS cells, Saos-2 and U2OS, was evaluated using flow cytometry. MSNs-HA_75_ exhibited superior cell uptake by both Saos-2 and U2OS cells compared to MSNs-HA_300_ and MSNs-HA_1650_ after 24 and 72 h exposure, as observed by a significant peak shift in the flow cytometry histograms (Figures 2B and S2). Notably, MSNs-HA_75_ displayed a higher cell uptake by Saos-2 cells compared to U2OS cells at both time points (Figure 2B). Interestingly, the fluorescence intensity of OS cells decreased after 72 h of exposure to MSNs-HA_75_ compared to 24 h of exposure (Figure 2B). This may be due to intracellular nanoparticle degradation when exposed to acidic and/or redox environments within OS cells. Furthermore, similar fluorescence after 24 and 72 h of exposure to MSNs-HA_300_ and MSNs-HA_1650_ was observed. It is likely that the nanoparticle uptake reached a plateau within 24 h, consistent with previous studies.^34−36^ In summary, MSNs-HA_75_ demonstrated enhanced cell uptake efficacy by OS cells, outperforming the observed uptake levels of MSNs-HA_300_ and MSNs-HA_1650_. Consequently, HA with a MW of 75 kDa was selected for the remainder of the study.

### 3.2 Development of Se and Dox Dual-Loaded MSNs Coated with HA

**Figure 3:**
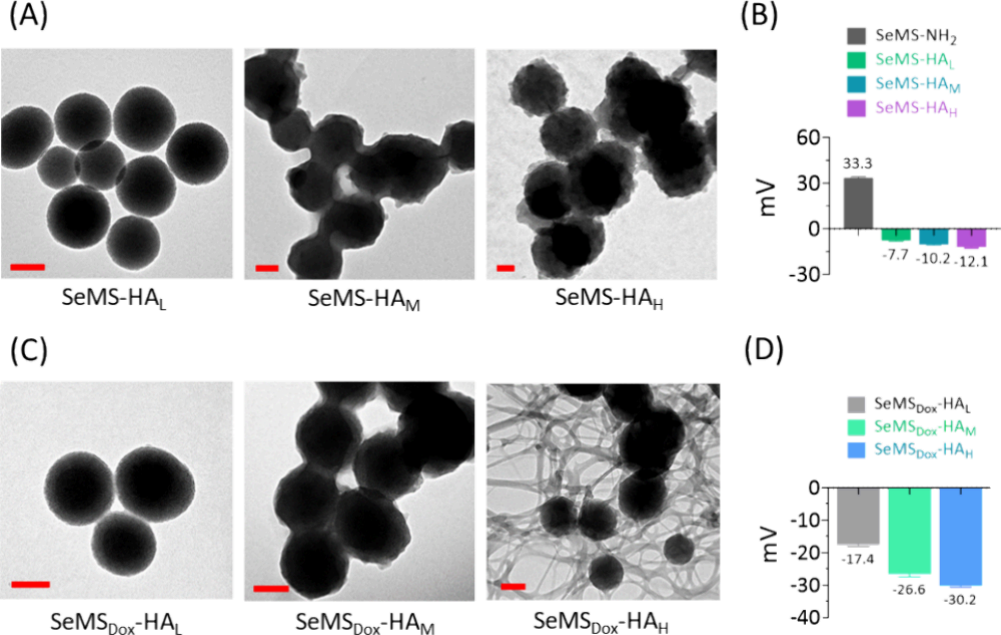
(A) TEM images of SeMS-HA_L_, SeMS-HA_M_, and SeMS-HA_H_; the scale bar is 100 nm. SeMS-HA_L_, HA_M_, and HA_L_ represent SeMS coated with various ratios of HA, being 1:0.05, 1:0.1, and 1:0.2 (SeMS:HA, wt/wt) respectively. (B) Surface charge of SeMS-NH_2_, SeMS-HA_L_, SeMS-HA_M_, and SeMS-HA_H_ in water (pH 7.4). (C) TEM images of SeMS_Dox_-HA_L_, SeMS_Dox_-HA_M_, and SeMS_Dox_-HA_H;_ scale bar is 100 nm. (D) Surface charge of SeMS_Dox_-HA_L_, SeMS_Dox_-HA_M_, and SeMS_Dox_-HA_H_ in water (pH 7.4). Data are presented as mean ± standard deviation (*n* = 3).

Next, SeMS were synthesized using our previously published one-pot method.^29^ Subsequently, SeMS was surface-modified by HA to form SeMS-HA. TEM analysis revealed a homogeneous spherical structure of the nanoparticles, with a mean particle size of approximately 155 nm (Figures S3 and 3A). The surface of SeMS-HA appeared blurry with less visible mesopores compared to SeMS, indicative of successful HA coating on the SeMS surface (Figures S3 and 3A). To further optimize the HA coating, we synthesized SeMS-HA with three different amounts of HA (w/w), 1:0.05, 1:0.1, and 1:0.2 (SeMS:HA, Table 1). Notably, SeMS-HA_H_ exhibited a blurrier surface compared to SeMS-HA_L_ and SeMS-HA_M_ (Figure 3A). The SeMS-HA size slightly increased when higher HA amounts were used, suggesting the formation of a thicker HA layer on the SeMS (Figure S4A). HA addition induced a shift in the SeMS-HA surface charge from positive to negative, validating the presence of the HA coating (Figure 3B). The nanoparticles were further characterized using FTIR. Characteristic peaks of both MS and HA were detectable in the FTIR spectra, confirming the presence of the silica matrix and functional groups of HA (Figure S4B). Specifically, bands at 449 cm^–1^ (Si–O–Si bending vibration), 798 cm^–1^ (Si–O–Si symmetric vibration), and 1090 cm^–1^ (Si–O–Si asymmetric vibration) were observed originating from the silica matrix.^37^ Distinct absorption peaks at 1607, 1403, and 1152 cm^–1^ could be attributed to the C=O, C–O, and C–O–C stretching modes of HA and were evident in all three nanoparticle formulations.^33^ Additionally, a peak at 1034 cm^–1^ was visible in the spectrum of SeMS-HA_H_, which can be attributed to the C–OH of HA, suggesting complete occupation of −COOH by −NH_2_. This peak was not visible when lower amounts of HA were used.

Dox loading in the silica mesopores was achieved by immersing SeMS in a Dox solution prior to HA coating. Initially, we investigated the effect of SeMS_Dox_ washing after Dox loading and showed a significant reduction in the Dox amount inside nanoparticles (Figure S5). Therefore, the HA coating was performed without nanoparticle washing in subsequent preparations. SeMS_Dox_-HA with different amounts of HA were prepared to investigate the effect of HA coating thickness on Dox release. The SeMS_Dox_-HA were characterized using TEM and DLS to assess morphology and surface modification, respectively. SeMS_Dox_-HA_L_ exhibited a morphology that was similar to that of SeMS (Figures 3C and S3). However, SeMS_Dox_-HA_M_ and SeMS_Dox_-HA_H_ displayed a thicker HA layer on the surface compared to that of SeMS_Dox_-HA_L_ (Figure 3C). SeMS_Dox_-HA_H_ revealed dense network formation, plausibly resulting from HA polymerization with unloaded Dox (Figure 3C). Overall, the morphology changes, negative surface charges, and fluorescence decrease of FITC in SeMS_Dox_-HA confirmed the HA coating on the surface of Dox-loaded SeMS (Figures 3C, D and S6). Also, MSNs loaded with Dox but without Se present in the core (MS_Dox_-HA) were synthesized to investigate the effect of Dox loading alone. For these preparations, medium and high ratios of HA were used to create MS_Dox_-HA_M_, and _H_, respectively. The blurry surface appearance and negative surface charge of MS_Dox_-HA revealed that the HA coating was successful (Figure S7).

### 3.3 Dox and Se Nanoparticle Loading and Release Profiles

**Figure 4:**
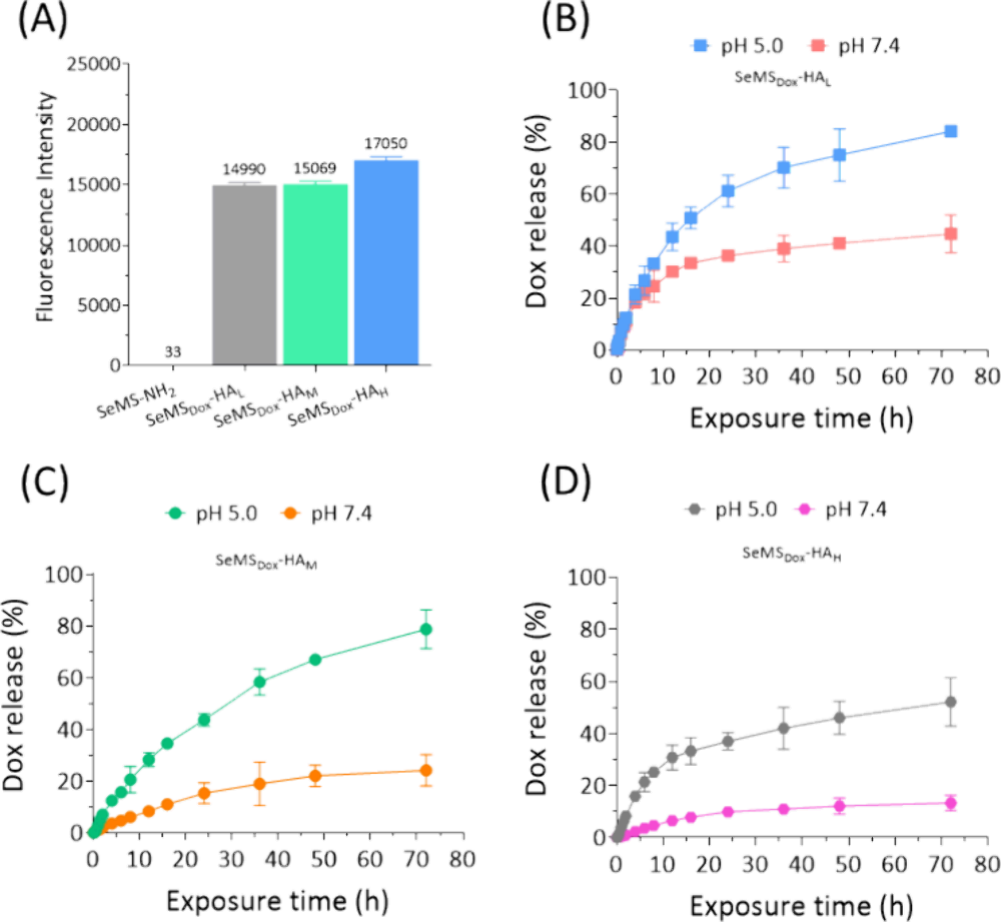
(A) Fluorescence intensity of SeMS_Dox_-HA_L_, SeMS_Dox_-HA_M_, and SeMS_Dox_-HA_H_ in water (pH 7.4). (B–D) Dox release (%) profile of SeMS_Dox_-HA_L_, SeMS_Dox_-HA_M_, and SeMS_Dox_-HA_H_ in pH 7.4 and 5.0 buffer after 72 h. All data are presented as mean ± standard deviation (*n* = 3).

Next, Dox loading efficiency in SeMS-HA and MS-HA was assessed by fluorescence intensity measurements (Figures 4A and S8A). Using Dox standard curves in pH 7.4 (Figure S9), and eqs 1 and 2 (methods section 2.5), the Dox content and loading efficiency for each group were determined (Table 2). Similar Dox content and loading efficiencies were observed for SeMS_Dox_-HA_L_, _M_, and _H_. Additionally, MS_Dox_-HA exhibited higher Dox content and loading efficiency compared to SeMS_Dox_-HA, possibly due to the larger volume of the mesopores in MS compared to SeMS, as reported previously.^29^ In that study, we showed that even though the pore diameters of MSNs and SeMS are similar, MSNs showed a significantly larger pore volume than SeMS.

**Table 2:** Dox Content (μg/mg) and Loading Efficiency (%) in Each Nanoparticle Group

sample code	dox content (μg/mg)	dox loading efficiency (%)
SeMS_Dox_-HA_L_	93.5 ± 1.4	26.7 ± 4.1
SeMS_Dox_-HA_M_	94.1 ± 0.4	26.9 ± 1.1
SeMS_Dox_-HA_H_	108.0 ± 1.4	30.9 ± 4.0
MS_Dox_-HA_M_	122.3 ± 1.8	34.9 ± 5.2
MS_Dox_-HA_H_	129.7 ± 1.0	37.1 ± 2.9

Dox release from the SeMS_Dox_-HA in neutral and acidic pH was determined (Figures 4B, D and S8B,C). Increased Dox release was observed at pH 5, where 61, 43, and 37% of total Dox release was observed for SeMS_Dox_-HA_L_, _M_, and _H_, respectively. This was significantly higher than the observed Dox release in neutral pH (Figure 4B, D, Table 3). Similarly, MS_Dox_-HA showed higher Dox release at pH 5.0 compared to pH 7.4 (Figure S8B,C). These results indicate pH-sensitive Dox release due to the HA coating on the nanoparticle surface. Moreover, a thicker HA coating led to slower Dox release under acidic conditions (Figures 4B,D and S8B,C).

**Table 3:** Dox Release (%) and Total Dox Release (μg/mg Nanoparticles) for Each Nanoparticle Group after 24 and 72 h Incubation in Neutral and Acidic Conditions

sample code	incubation time (h)	dox release (%)	
		pH 5.0	pH 7.4
SeMS_Dox_-HA_L_	24	61.2 ± 6.0	36.2 ± 1.0
	72	84.1 ± 0.3	44.7 ± 7.3
SeMS_Dox_-HA_M_	24	43.7 ± 2.4	15.4 ± 4.0
	72	78.1 ± 7.4	24.2 ± 6.0
SeMS_Dox_-HA_H_	24	37.0 ± 3.3	9.9 ± 0.1
	72	52.1 ± 9.3	13.3 ± 3.0

**Figure 5:**
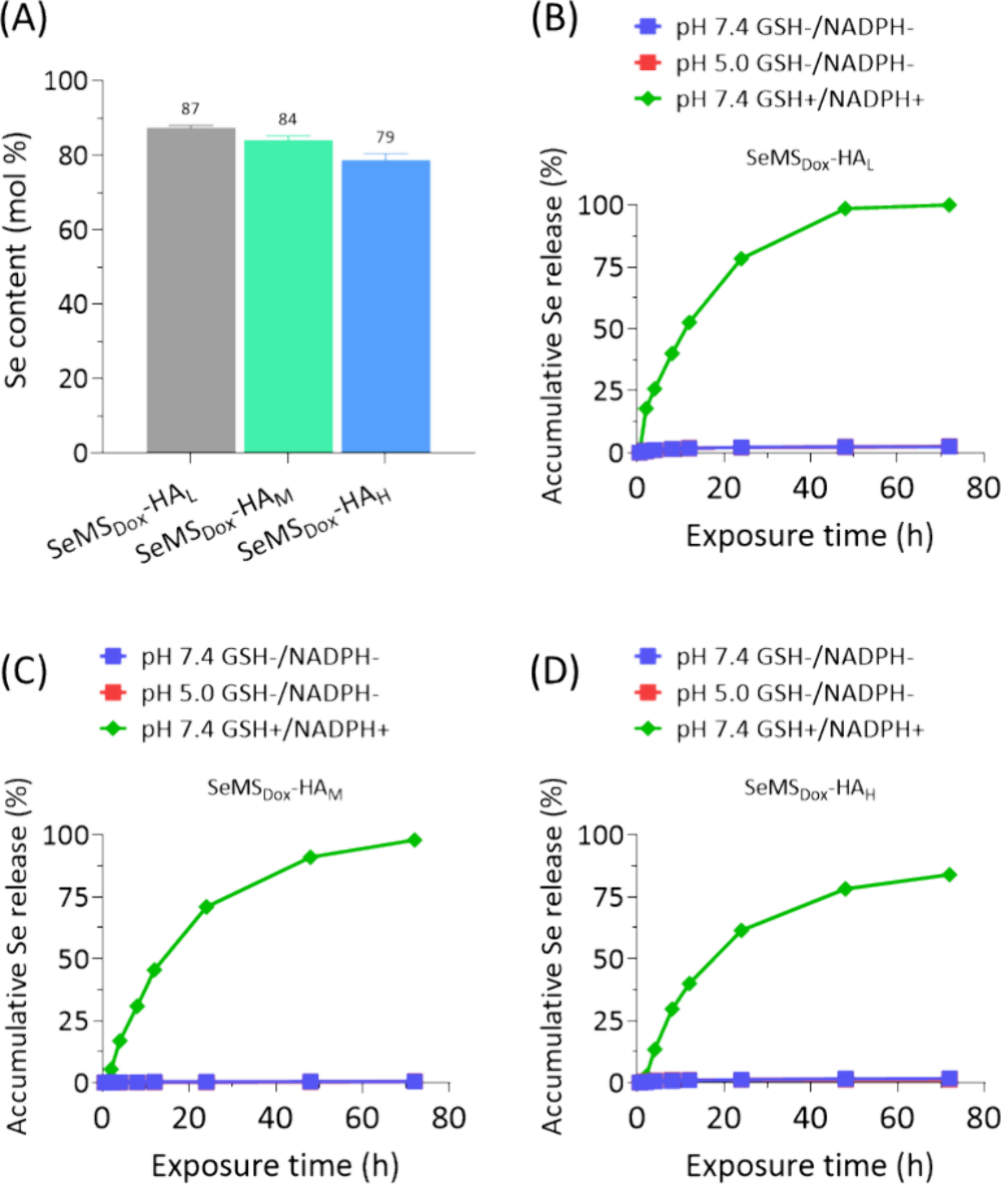
(A) Se content (mol %) in SeMS_Dox_-HA_L_, SeMS_Dox_-HA_M_, and SeMS_Dox_-HA_H_ measured by ICP-MS. (B–D) Cumulative Se release profile (%) of SeMS_Dox_-HA_L_, SeMS_Dox_-HA_M_, and SeMS_Dox_-HA_H_ (125 μg/mL) at pH 7.4 (±GSH/NADPH) and 5.0. All data are presented as mean ± standard deviation (*n* = 3).

Next, Se loading and release from the nanoparticles were evaluated using ICP-MS analysis. To investigate Se release, SeMS_Dox_-HA was dispersed in cacodylate buffer with either neutral (pH 7.4), acidic (pH 5.0), or neutral pH containing GSH/NADPH+, to mimic the extracellular and intracellular surroundings of OS cells, respectively. SeMS_Dox_-HA_L_, SeMS_Dox_-HA_M_, and SeMS_Dox_-HA_H_ contained a high Se content, ranging between 79 and 87 mol % (Figure 5A), which is consistent with our previous findings.^29^ Notably, under redox conditions, over 80% Se release was observed for SeMS_Dox_-HA_L_, SeMS_Dox_-HA_M_, and SeMS_Dox_-HA_H_, after 3 days of incubation. In contrast, less than 5% Se release was observed in neutral and acidic pH values (Figure 5B–D). This is in line with the literature; there are several reports demonstrating that SeNP degrades in redox environments.^16,29,38−40^ Moreover, SeMS_Dox_-HA_L_ showed significantly faster and higher Se release compared to the other two nanoparticles, implying that HA thickness influences the Se release rate (Figure 5B–D).

In conclusion, SeMS_Dox_-HA could efficiently load Dox and Se. Moreover, pH and redox responsive Dox release was demonstrated. Se release was redox-responsive.

In summary, based on the previous findings from TEM, DLS, Dox/Se loading, and release, SeMS_Dox_-HA_M_ was selected for further in vitro studies, as it showed more homogeneous morphology compared to SeMS_Dox_-HA_H_ and lower Dox leakage compared to SeMS_Dox_-HA_L_.

### 3.4 Cellular Uptake in OS Cells

**Figure 6:**
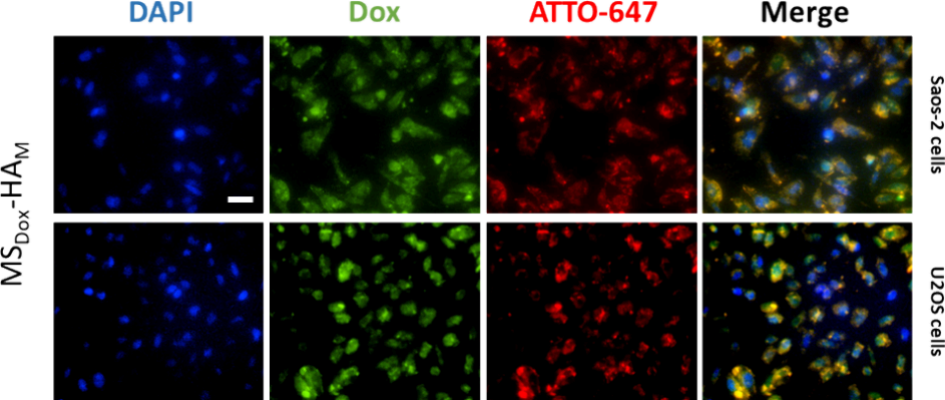
Intracellular uptake of MS_Dox_-HA_M_ (100 μg/mL) in Saos-2 and U2OS cells after 6 h of incubation; scale bar is 50 μm. Nuclei were stained with DAPI (blue channel). The green channel indicates loaded Dox. The red channel indicates ATTO-647 dye labeling in MS_Dox_-HA.

MSNs are known to be efficiently taken up by OS cells via endocytosis within 6 h,^41−43^ facilitating intracellular delivery of therapeutics.^44^ To verify that the nanoparticles can deliver Dox for intracellular release, the cell uptake of nanoparticles was investigated by exposing OS cells to Se/Dox-dual loaded MSNs (SeMS_Dox_-HA_M_) and Dox only loaded MSNs (MS_Dox_-HA_M_) by using fluorescent microscopy. Dox is naturally fluorescent (λ_ex_ = 480 nm and λ_em_ = 560 nm; here shown as green) and can be used to track drug release from the nanoparticles. MS_Dox_-HA_M_ could be modified with thiol groups in the core of the particles to allow attachment of ATTO-647 dye, enabling us to track MSNs in cells. After 6 and 12 h of incubation, colocalization of Dox and ATTO-647 was observed, demonstrating that nanoparticles were internalized into OS cells along with Dox (Figures 6 and S10). Thus, these results indicate that Dox is transported into OS cells via MSN cell internalization rather than through passive diffusion of extracellularly released Dox. This also indicated that MS_Dox_-HA_M_ could be readily internalized by both Saos-2 and U2OS cells within a short time frame. Additionally, Dox fluorescence was observed in OS cells after exposure to SeMS_Dox_-HA_M_ after 6 and 12 h of incubation. Given the similar surface properties of SeMS_Dox_-HA_M_ compared to MS_Dox_-HA_M_, it is likely that SeMS_Dox_-HA_M_ can also be uptaken by OS cells (Figure S11). In summary, our findings confirm that Dox-loaded nanoparticles can deliver Dox into cells within 6 h.

### 3.5 SeMS_Dox_-HA_M_ Cytotoxicity in OS Cells and hMSCs

**Figure 7:**
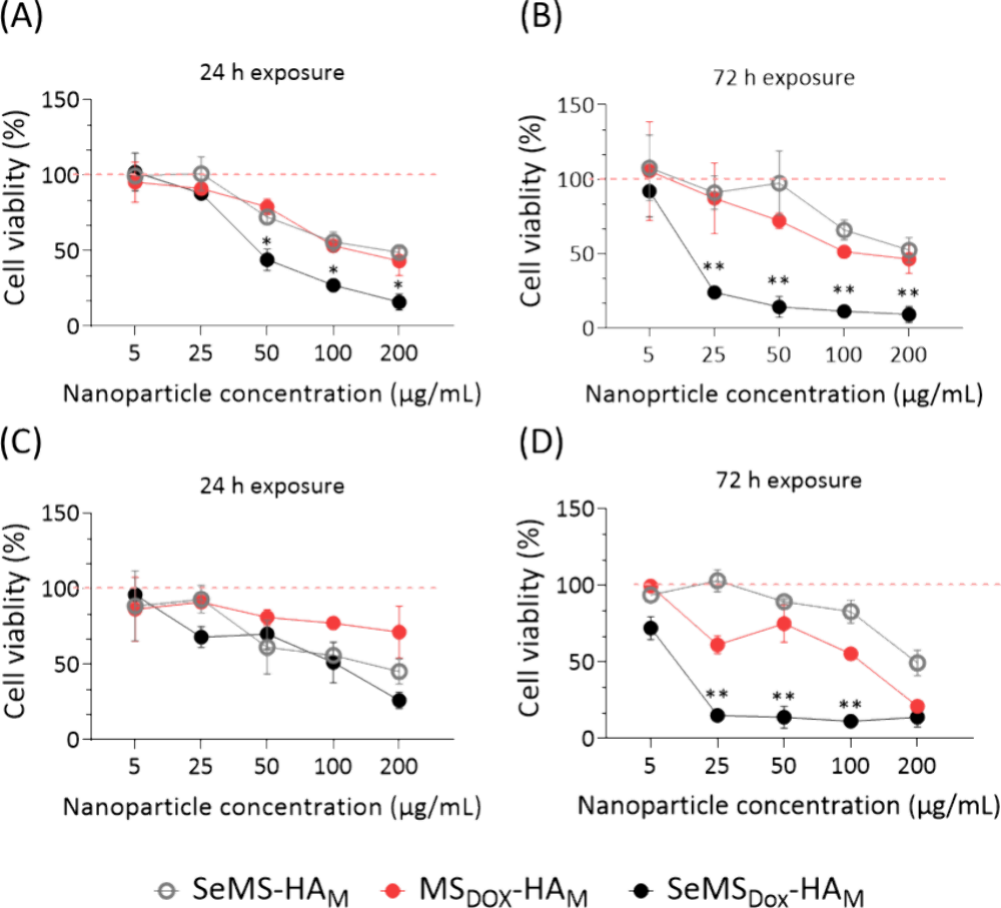
Cell viability of Saos-2 incubated with SeMS-HA_M_, MS_Dox_-HA_M_, and SeMS_Dox_-HA_M_ for (A) 24 and (B) 72 h. Cell viability of U2OS incubated with SeMS-HA_M_, MS_Dox_-HA_M_, and SeMS_Dox_-HA_M_ for (C) 24 h and (D) 72 h. All data are presented as mean ± standard deviation (*n* = 3). * presents statistical significance (*: *p* < 0.005; **: *p* < 0.0001).

Next, we assessed the synergistic impact of Se and Dox, administered via our MSN delivery system, on the metabolic activity of OS cells using the MTS assay. A decrease in metabolic activity is used as an indirect measure of their cytotoxicity. The inhibitory effect of SeMS-HA_M_, MS_Dox_-HA_M_, and SeMS_Dox_-HA_M_ on OS cell lines Saos-2 and U2OS was investigated after 24 and 72 h exposure. Dose-dependent toxicity upon exposure to the nanoparticles was observed in both OS cell lines (Figure 7). The nanoparticle concentrations that induced 25% (IC_25_), 50% (IC_50_), and 75% (IC_75_) cell viability loss are shown in Table 4. Particularly noteworthy was the cytotoxic effect of SeMS_Dox_-HA_M_ on Saos-2 cells, with IC_50_ values of 39.5 (24 h) and 6.2 μg/mL (72 h). These were significantly lower than the SeMS-HA_M_ and MS_Dox_-HA_M_ IC_50_ values (Table 4). Moreover, SeMS_Dox_-HA_M_ showed increased cytotoxicity toward Saos-2 cells after a longer exposure time of 72 h (Figure 7A, B), probably due to the gradual degradation of HA and release of Dox/Se. Also in U2OS cells, SeMS_Dox_-HA_M_ demonstrated a low IC_50_ value of 2.5 μg/mL after 72 h exposure that was significantly higher than the IC_50_ values of either treatment alone. (Table 4). Specifically, after 72 h exposed to 50 μg/mL SeMS_Dox_-HA_M,_ an 86% reduction in U2OS cell metabolic activity was measured, whereas SeMS-HA_M_ and MS_Dox_-HA_M_ only resulted in an 11 and 25% reduction, respectively (Figure 7C, D). Thus, our findings indicate that the codelivery of Dox and Se through an MSN delivery system represents a viable strategy to enhance the cytotoxic efficacy of both agents.

**Table 4:** IC_25_, IC_50_, and IC_75_ Values of Saos-2 and U2OS Cells Incubated with SeMS-HA_M_, MS_Dox_-HA_M_, and SeMS_Dox_-HA_M_ for 24 and 72 h^a^

cell type	incubation time (h)	samples	IC_25_ (μg/mL)	IC_50_ (μg/mL)	IC_75_ (μg/mL)
Saos-2	24	SeMS-HA_M_	38.2	47.2	58.4
		MS_Dox_-HA_M_	45.8	66.5	96.7
		SeMS_Dox_-HA_M_	28.0	39.5	55.7
	72	SeMS-HA_M_	68.9	84.8	104.6
		MS_Dox_-HA_M_	23.1	43.7	82.9
		SeMS_Dox_-HA_M_	3.0	6.2	12.7
U2OS	24	SeMS-HA_M_	45.3	47.7	50.2
		MS_Dox_-HA_M_	45.6	69.8	106.7
		SeMS_Dox_-HA_M_	/	/	/
	72	SeMS-HA_M_	200.7	351.7	616.4
		MS_Dox_-HA_M_	/	/	/
		SeMS_Dox_-HA_M_	1.5	2.5	4.2

a Note: “/” means the IC_25_, IC_50_, or IC_75_ values cannot be measured.

Also, potential cytotoxic effects of SeMS_Dox_-HA_M_ toward hMSCs were investigated after 24 and 72 h exposure. Notably, SeMS_Dox_-HA_M_ showed low toxicity toward hMSCs after 24 h up to 50 μg/mL (Figure S12). After 72 h, at relatively high dosages (≥50 μg/mL), a significant impact on cell viability was observed (Figure S12). Interestingly, SeMS_Dox_-HA_M_ showed significantly higher cytotoxicity toward OS compared to hMSCs, at concentrations of 25 μg/mL and below.

### 3.6 ROS Modulation in OS Cells

**Figure 8:**
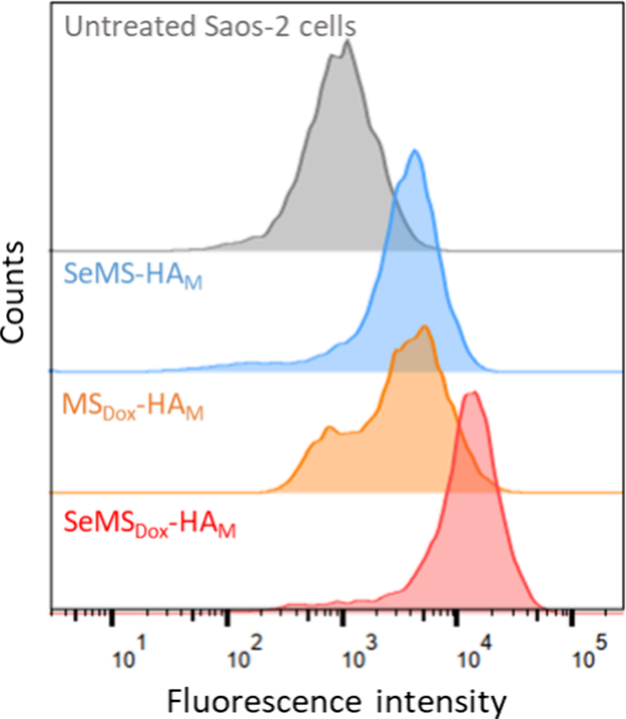
Flow cytometry curves showing ROS levels (via DCF/DA staining) in Saos-2 cells after 24 h of exposure to 100 μg/mL SeMS-HA_M_, MS_Dox_-HA_M_, and SeMS_Dox_-HA_M_. All data are presented as mean ± standard deviation (*n* = 3).

Se and Dox both have known ROS-inducing capabilities,^8,16^ which have been used to induce cell apoptosis that is more selective toward cancer cells compared to normal cells.^45,46^ To investigate whether the release of Dox and Se from our nanoparticles could promote ROS production, OS cells were exposed to SeMS-HA_M_, MS_Dox_-HA_M_, and SeMS_Dox_-HA_M_ for 24 h followed by staining with the DCFH/DA probe. Notably, all three nanoparticles induced ROS production in Saos-2 cells, where SeMS that delivered both Dox and Se (i.e., SeMS_Dox_-HA_M_) were most effective (Figure 8). Our findings thus demonstrate that combined Dox and Se delivery using SeMS can lead to significantly increased ROS production in OS cells. Similar ROS levels were observed in OS cells treated with either SeMS-HA_M_ or MS_Dox_-HA_M_, indicating that Dox and Se had similar ROS-inducing capabilities when delivered as monotherapies. However, a smaller peak was observed in the histogram of OS cells treated with MS_Dox_-HA_M_, potentially due to differences in the mechanism of ROS induction compared to Se. In summary, our findings demonstrate that Se/Dox-dual delivery using SeMS is an effective method to induce ROS production in OS cells.

## 4 Discussion

Dox is used as a chemotherapeutic agent in OS therapy and acts by interfering with DNA replication.^47^ However, the efficiency of Dox-based therapeutics is hindered by low specificity and poorly controlled drug delivery, which can lead to severe side effects.^48^ Moreover, continuous Dox treatment can lead to cancer cell resistance, further reducing its therapeutic efficacy.^49^ CT facilitated by the use of nanoparticle carriers is a promising strategy to overcome the issues associated with Dox use. Combining Dox with other therapeutics can enhance its anti-OS efficiency and overcome cancer cell resistance.^50^ In this regard, Se seems to be a suitable candidate for CT with Dox since Se can induce the generation of ROS, leading to OS cell apoptosis.^51^ Nanoparticles can be used to improve CT effectiveness by encapsulating multiple therapeutics such as Se and Dox within a single nanocarrier for delivery.^52^ For instance, Se-doped calcium phosphate nanoparticles loaded with Dox enhanced OS chemotherapy via reversing multidrug resistance.^53^ In this work, we used MSNs to facilitate CT of Se/Dox toward OS cells. While there are several studies investigating the codelivery of Se and Dox in nanoparticles such as calcium phosphate,^53−56^ the use of MSNs for codelivery of Se and Dox has not been explored. The use of MSNs can present benefits over other nanoparticles due to their biocompatibility, large drug loading volume, protective structure for dual-therapeutic encapsulation, and possibility for controlled drug release.^57−59^ In this study, we report the successful development of Dox and SeNP incorporation in MSNs followed by HA coating for pH-responsive drug delivery to OS cells.

We initially investigated the effect of the MW of HA on the OS cell nanoparticle uptake efficiency. MSNs coated with low MW HA (75 kDa) demonstrated the highest OS cell uptake compared to middle (300 kDa) and high MW HA (1650 kDa). This increase in cell uptake can be attributed to the higher binding affinity of HA toward CD44 receptors, which are known to be overexpressed on cancer cells, including OS cancer cells.^60^ The influence of HA MW on CD44 receptor binding has been reported in various studies.^61−66^ Contrary to some reports suggesting that either HA with higher MW (400–1000 kDa) or HA oligosaccharides (<10 kDa) exhibit superior binding to CD44,^61,62,67^ our findings aligned with studies suggesting the 50–200 kDa HA MW range as optimal for CD44 binding.^64−66^ These differences may be attributed to the different cell types that were used in these studies, such as Hela, MCF-7, or MDA-MB-231 cells.^63−66^ Importantly, our study was the first to explore the effect of the MW of HA on OS cell nanoparticle uptake.

Although Dox encapsulation was successful, when high amounts of HA were used_,_ network formation among and around the nanoparticles could be observed. This is likely due to an interaction between unloaded Dox and exceeded amounts of HA since this network formation was not observed when Dox was not present. When HA with medium thickness was used to create SeMS_Dox_-HA_M_, no network formation was observed and a Dox loading amount of 94.1 μg/mg was achieved, which is comparable to Dox-loaded MSNs as reported in other studies.^68,69^ Sustained Dox release was observed which was higher under acidic conditions. This can be attributed to the degradation of the HA coating at lower pH values; HA undergoes faster hydrolysis in acidic conditions by breaking glycosidic bonds.^25^ Consequently, in our nanoparticle system, the mesopores were gradually exposed after HA glycosidic bond cleavage, facilitating Dox release. The HA coating thickness affected the extent of Dox release under both acidic and neutral conditions. The nanoparticles with the thickest HA coating, i.e. SeMS_Dox_-HA_H_, exhibited the slowest Dox release. Conversely, the thinnest HA coating (SeMS_Dox_-HA_L_) resulted in fast Dox release, even at neutral pH. The pH values 7.4 and 5.0 used in our release study resemble the extracellular matrix and lysosome environment, suggesting that SeMS_Dox_-HA can facilitate a rapid Dox release upon entering cells while remaining stable in the extracellular environment.

SeMS_Dox_-HA induced a higher cytotoxicity in OS cells compared to either SeMS-HA or MS_Dox_-HA, indicating a synergistic effect of intracellularly released Dox and Se. Specifically, SeMS_Dox_-HA_M_ demonstrated over a 2-fold increase in OS cell viability loss compared to MS_Dox_-HA_M_ or SeMS-HA after 72 h, even though MS_Dox_-HA_M_ contained a higher Dox amount (122.3 μg/mg) than SeMS_Dox_-HA_M_ (94.1 μg/mg). It is worth noting that both SeMS_Dox_-HA and MS_Dox_-HA were internalized by OS cells to a similar extent. Recent studies have demonstrated that both Dox and Se can elevate ROS levels, leading to mitochondrial apoptosis.^8,16^ ROS can induce apoptosis in OS cells when its concentration exceeds a critical threshold.^15^ The combination of Dox and Se in SeMS_Dox_-HA may accelerate ROS-mediated apoptosis, enhancing the cytotoxicity. Our ROS data supports this hypothesis, showing significantly higher ROS levels in OS cells treated with the CT compared to Dox or Se alone. Therefore, our findings suggest that utilizing an MSN-based delivery system for the combined administration of Dox and Se can effectively increase ROS production, potentially leading to more selective OS therapy.

While the Dox/Se dual-loaded MSNs show promise in delivering multiple therapeutic agents while also enhancing their intracellular drug release, several limitations and challenges need to be addressed for future applications. These include careful dosing and the potential toxicity of Se. Although we and others have shown that Se can induce toxicity in OS cells via ROS induction, this is highly dependent on dose, forms of Se (e.g., as an ion, compound, or nanoparticle^22,29,40^), and release profile. In our system, the HA layer must first degrade to release Dox, followed by Se nanoparticle degradation under redox conditions. This adds complexity as we must ensure both drugs are released in a controlled and synergistic manner in order to not compromise therapeutic efficacy. Additionally, as our results suggest, the variability in cell response may also depend on the type and condition of cancer cells. This heterogeneity can affect treatment outcomes, highlighting the need for further studies to better understand how different cancer cell lines and tumor microenvironments respond to our Dox/Se dual-loaded nanoparticle system.

## 5 Conclusions

In this study, we developed Dox/Se dual-loaded mesoporous silica nanoparticles (SeMS_Dox_-HA) as a novel approach for combination OS therapy. We successfully optimized the incorporation of Dox and Se into the MSNs by adjusting the MW of HA and the coating thickness. Our findings demonstrate that HA with a molecular weight of 75 kDa exhibited the highest uptake efficiency by OS cells in vitro. Moreover, the thickness of the HA coating was found to significantly influence the release rate of Dox and its stability, with a medium coating thickness proving to be the most effective in maintaining stability at neutral pH and facilitating drug release at acidic pH. The dual treatment combining Dox and Se, delivered through HA-coated MSNs, was more effective at inhibiting OS cell viability in vitro than either Dox or Se administered alone, which may be attributed to more effective ROS upregulation. Overall, the Dox/Se dual-loaded MSNs developed in this work serve as a compelling example of the potential of using Dox and Se in CT, in synergy with MSNs as nanodelivery vehicles. Moreover, this study provides some important design rules for optimizing and further development of these nanoparticles. Dox-loaded SeMS holds significant promise for application in both systemic and local OS therapy, particularly when combined with bone graft substitutes for localized OS treatments.

## Supporting Information Available

Methods of MSNs, SeMS (Schemes S1 and S2), SeMS_Dox/W_-HA synthesis and characterization, and cell culture; Se cumulative release model (Scheme S3); MSNs-HA characterization (Figure S1); flow cytometry analysis of nanoparticle cell uptake (Figure S2); TEM images of SeMS (Figure S3); SeMS-HA and SeMS_Dox/W_-HA characterization (Figures S4 and S5); fluorophore labeling analysis (Figure S6); MS_Dox_-HA characterization (Figure S7); Dox loading and release profiles (Figure S8); Dox standard curves (Figure S9); fluorescence microscopy images of nanoparticle cell uptake (Figures S10 and S11); and metabolic activity assay (Figure S12) (PDF)
