# Supplementary material for: Doxorubicin- and Selenium-Incorporated Mesoporous Silica Nanoparticles as a Combination Therapy for Osteosarcoma
Source: ACS Appl Nano Mater. 2024 Nov 1;7(22):25400–11. doi: 10.1021/acsanm.4c04294 (PMC11590048; doi:10.1021/acsanm.4c04294)
Supplement: Supplementary file 1 — an4c04294_si_001.pdf [file an4c04294_si_001.pdf]

Supporting Information

Doxorubicin- and Selenium-Incorporated Mesoporous Silica Nanoparticles as a Combination Therapy for Osteosarcoma

Lei He, Zahra Javid Anbardan, Pamela Habibovic and Sabine van Rijt*

Department of Instructive Biomaterials Engineering, MERLN Institute for Technology Inspired Regenerative Medicine, Maastricht University, P.O. Box 616, 6200 MD Maastricht, The Netherlands.

**Corresponding author. E-mail: s.vanrijt@maastrichtuniversity.nl*

1. Methods

1.1 Synthesis of –SH/–NH_2 functionalized mesoporous silica nanoparticles (MSNs)

Initially, tetraethyl orthosilicate (TEOS; 1.747 mL), 3-mercaptopropyl triethylsilane (MPTES; 0,114 mL), and triethanolamine (TEA; 14.3 g) were combined to create *Solution-1*. Concurrently, cetyltrimethylammonium chloride (CTAC 25 wt% in H_2O; 2.41 mL), ammonium fluoride (NH_4F; 100 mg), and H_2O (milliQ water; 21.7 mL) formed *Solution-2*. *Solution-1* was heated statically in oil bath at 90 °C, while *Solution-2*, under continuous stirring (500 rpm), was heated at 60 °C. After 20 min, *Solution-2* was rapidly poured into *Solution-1* under vigorous stirring (1000 rpm) for an additional 20 min at room temperature. TEOS (0.148 mL) was incrementally added in four equal parts (37 µL) every 3 min, and the mixture was stirred for another 30 min at room temperature. A mixture of TEOS (20.7 µL) and 3-aminopropyl triethoxysilane (APTES; 0.2 mL) was then introduced into the emulsion for overnight stirring at room temperature. The resulting MSNs underwent two ethanol washes, followed by template removal through two rounds of reflux. The MSNs were heated sequentially in ammonium nitrate ethanoic solution (NH_4NO_3; 2 g in 100 ml ethanol) and hydrochloric acid (37% HCl) ethanoic solution at 90 °C for 45 min, respectively. Finally, the template-removed MSNs were collected, triple-washed with ethanol, and stored at 4 °C for subsequent use.

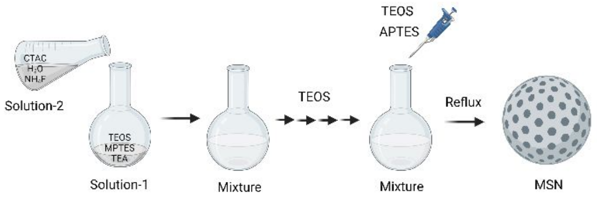

Scheme S1 Synthesis of MSN.

1.2 Synthesis of –NH_2 functionalized SeMS

Briefly, CTAB (0.36 g), ascorbic acid (Vc; 1 g), and NH_4F (0.6 g) were dissolved in 100 mL milliQ water while vigorously stirring (1200 rpm) at 80 °C. Na_2SeO_3 water solution (0.25 g/mL; 4.144 mL) was added dropwise to the mixture under continuous stirring (1200 rpm) at 80 °C for 1 h. Subsequently, TEOS (1.6794 g) was added dropwise with 30 min stirring at room temperature. The TEOS layer-by-layer assembly, amino- functionalization, and reflux steps were mirrored the MSN synthesis procedures.

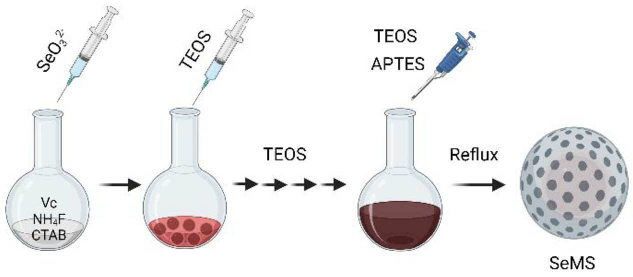

Scheme S2 Synthesis of SeMS.

1.3 Synthesis and characterization of $SeMS_{Dox/W}$-HA

20 mg SeMS resuspended in 3 mL milliQ water were mixed with 7 mL Dox solution (0.1 mg/mL) and stirred overnight, to produce Dox-loaded SeMS ($SeMS_{Dox}$). An additional washing step was introduced followed by the formation of $SeMS_{Dox}$, producing $SeMS_{Dox/W}$. $SeMS_{Dox/W}$ were reacted with middle (SeMS/HA=1:0.1, wt/wt) and high (SeMS/HA=1:0.2, wt/wt) ratios of HA and kept stirring overnight, collected, washed once with water, and stored in the freezer, labelled as $SeMS_{Dox/W}$-HA. Two variables, $SeMS_{Dox/W}$-HA_M and $_L$, represented nanoparticles coated with middle and high ratios of HA. Characterization of the $SeMS_{Dox/W}$-HA was performed using TEM and DLS to assess morphology and surface charge (zeta potentials, ζ). The Cary Eclipse Fluorescence Spectrophotometer (Agilent Technologies; US) was employed to measure the fluorescence of $SeMS_{Dox/W}$-HA (λ_{ex} = 480 nm and λ_{em} = 560 nm) [1].

1.4 Cell culture

Saos-2 and U2OS were selected as osteosarcoma (OS) cell lines for cell internalization studies and cell viability tests using MTS assay. Saos-2, exhibiting epithelial morphology, is derived from a female OS patient, while U2OS, an epithelial-like cell line, is derived from a 15-year-old female OS patient (ATCC, US). Cells were cultured in DMEM (high glucose, L-glutamine, Gibco, US) supplemented with 10% fetal bovine serum (FBS; Gibco, US), and 100 U/mL penicillin and streptomycin (P/S; Gibco, US) at 37 °C, 5% CO_2 (normal O_2 level).

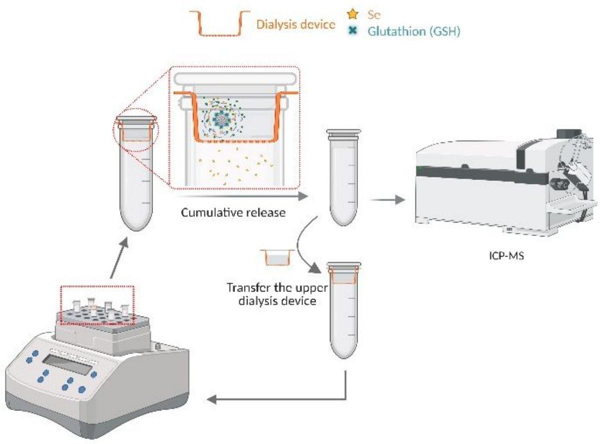

Scheme S3 Setup of cumulative Se release studies from the nanoparticles.

2. Results

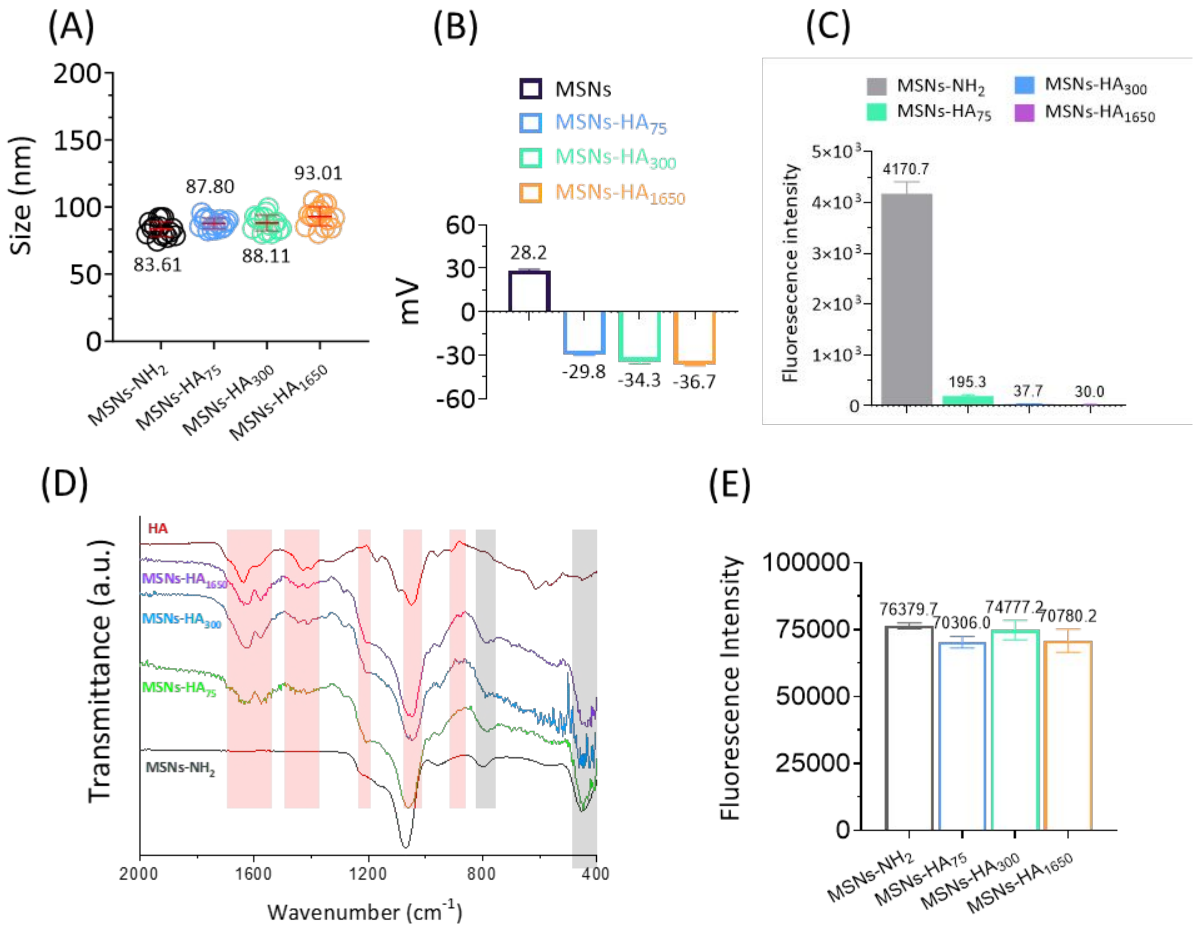

Figure S1 (A) Size distribution of MSNs-NH_2, MSNs-HA_{75}, MSNs-HA_{300}, and MSNs-HA_{1650} (n = 20). (B) Surface charge of MSNs-NH_2, MSNs-HA_{75}, MSNs-HA_{300}, and MSNs-HA_{1650}. (C) Fluorescence intensity of fluorophore (NHS-FTIC dye) labeled MSNs-NH_2, MSNs-HA_{75}, MSNs-HA_{300}, and MSNs-HA_{1650} to illustrate the –NH_2 group in the nanoparticles. (D) FTIR spectra of MSNs-NH_2, MSNs-HA_{75}, MSNs-HA_{300}, and MSNs-HA_{1650} (gray areas represented the peaks of MSNs and pink areas represented the peaks of HA). (E) Fluorescence intensity of dye labelled MSNs-NH_2, MSNs-HA_{75}, MSNs-HA_{300}, and MSNs-HA_{1650}. All data are presented as mean ± standard deviation (n=3).

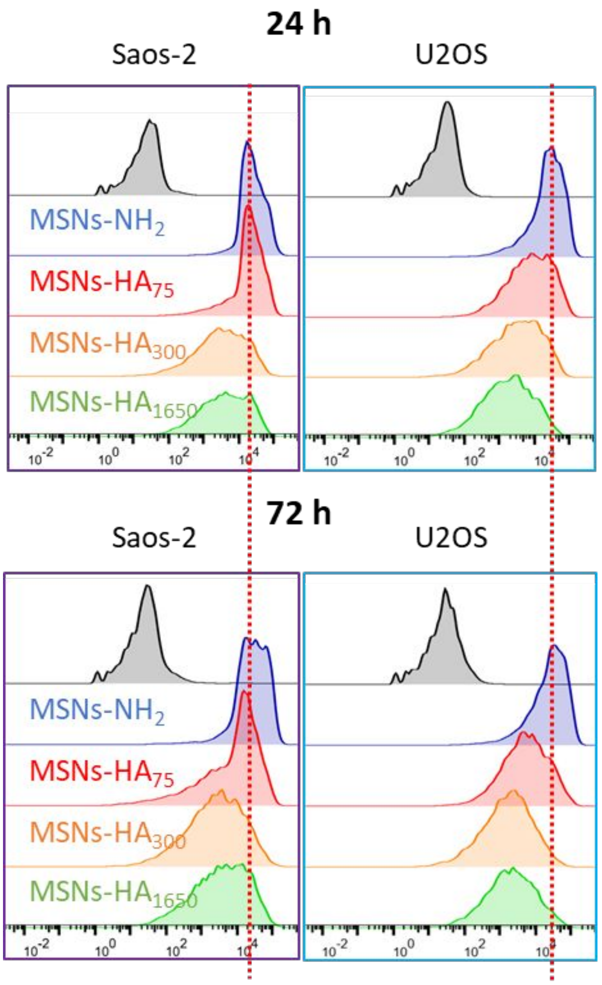

Figure S2 Cellular uptake of dye labelled MSNs-NH_2, MSNs-HA_{75}, MSNs-HA_{300}, and MSNs-HA_{1650} (200 µg/mL) on different cell types via flow cytometry after 24 and 72 h-exposure.

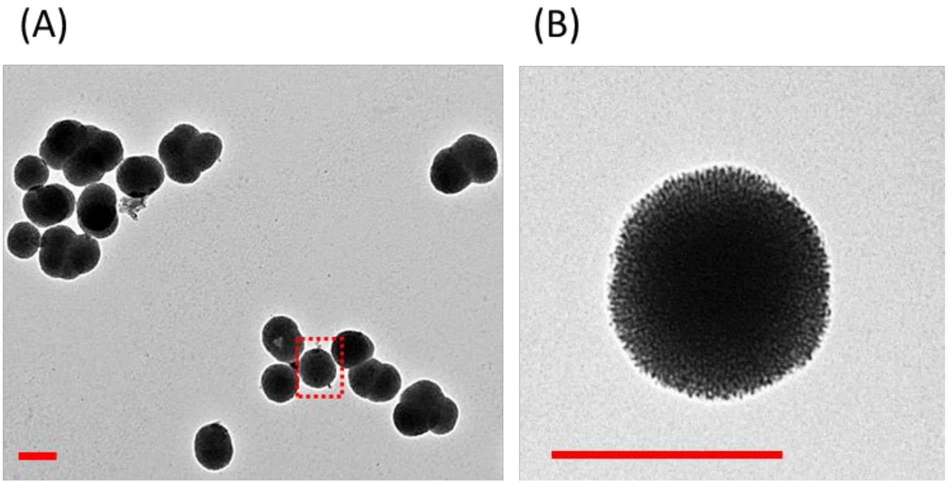

Figure S3 TEM images of SeMS-NH_2 in (A) low magnification and (B) high magnification; scale bars are 200 nm.

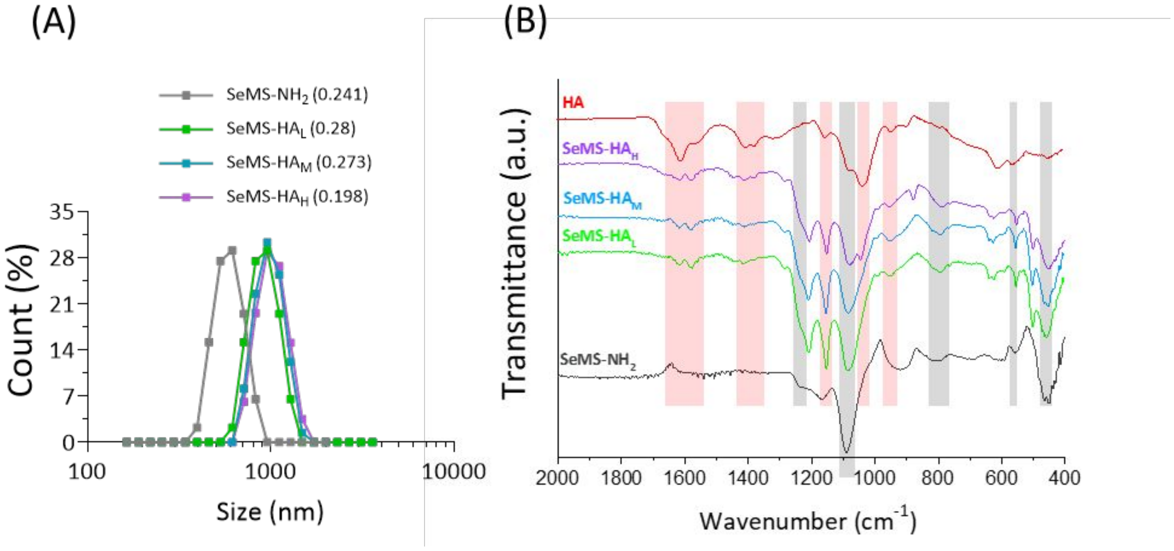

Figure S4 (A) Size distribution of SeMS-NH_2, SeMS-HA_L, SeMS-HA_M, and SeMS-HA_H via DLS in ethanol. (B) FTIR spectra of SeMS-NH_2, SeMS-HA_L, SeMS-HA_M, and SeMS-HA_H (gray areas represented the peaks of MS and pink areas represented the peaks of HA). (C) Fluorescence intensity of fluorophore (NHS-FTIC dye) labeled SeMS-NH_2, SeMS-HA_L, SeMS-HA_M, and SeMS-HA_H to illustrate the –NH_2 group in nanoparticles. All data are presented as mean ± standard deviation (n=3).

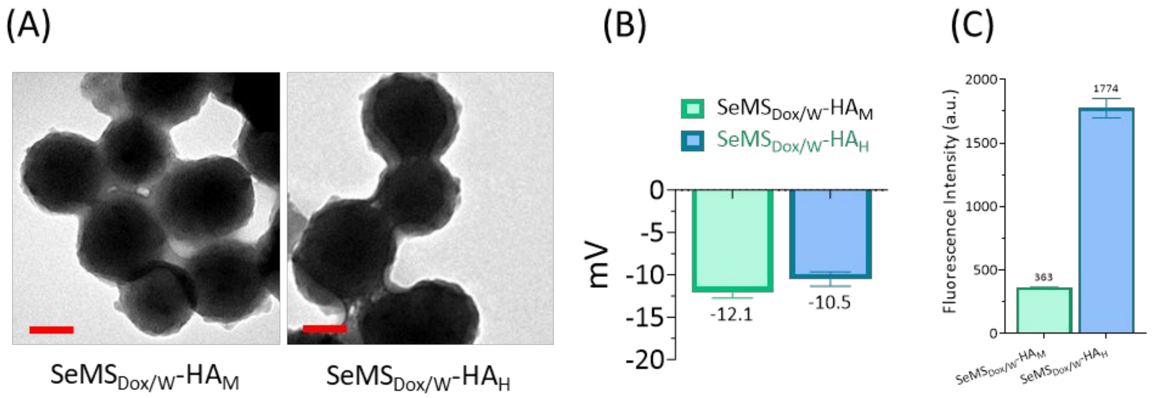

Figure S5 (A) TEM images of $SeMS_{Dox/W}$-HA_M and $SeMS_{Dox/W}$-HA_H; scale bar is 100 nm. HA_M and HA_L represented different amounts of coated HA on SeMS (B) Surface charge of $SeMS_{Dox/W}$-HA_M and $SeMS_{Dox/W}$-HA_H in water (pH 7.4). (C) Fluorescence intensity of $SeMS_{Dox/W}$-HA_M, and $SeMS_{Dox/W}$-HA_H in water (pH 7.4). Data are presented as mean ± standard deviation (n=3).

To investigate the effect of washing step after Dox loading procedure on the loading efficiency in SeMS-HA, $SeMS_{Dox/W}$-HA were synthesized. $SeMS_{Dox/W}$-HA_M and $_H$ displayed blurry surface and thicker layer on the surface compared to SeMS. Combining with the negative surface charges of $SeMS_{Dox/W}$-HA, both TEM and DLS results validated the presence of the HA coating on the surface of $SeMS_{Dox/W}$-HA (**Figure S5A-B**). However, after comparing the fluorescence intensity of $SeMS_{Dox}$-HA and $SeMS_{Dox/W}$-HA, significant lower fluorescence was

observed in $SeMS_{Dox/W}$-HA, indicated that the washing step resulted in a loss of Dox (**Figure S5C, Figure 4A**). Thus, $SeMS_{Dox}$-HA were selected for further subsequent studies.

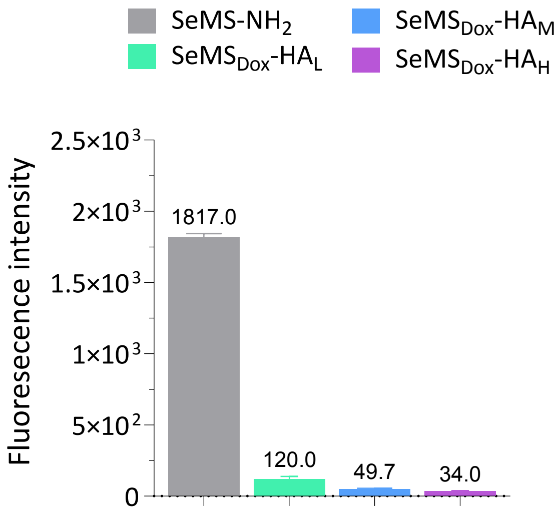

Figure S6 Fluorescence intensity of fluorophore (NHS-FTIC dye) labeled $SeMS-NH_2$, $SeMS-HA_L$, $SeMS-HA_M$, and $SeMS-HA_H$ to illustrate the $–NH_2$ group in nanoparticles. All data are presented as mean ± standard deviation (n=3).

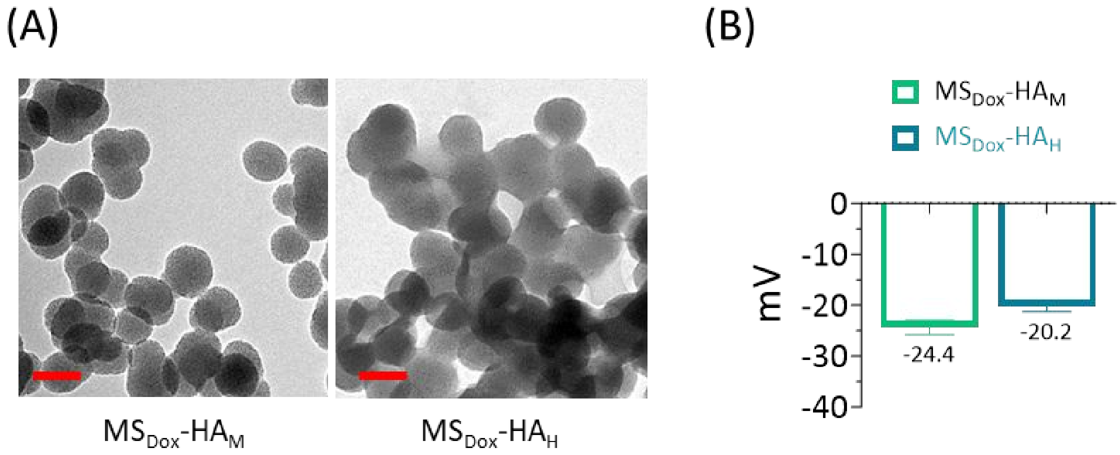

Figure S7 (A) TEM images of $MS_{Dox}-HA_M$ and $MS_{Dox}-HA_H$; scale bar is 100 nm. (B) Surface charge of $MS_{Dox}-HA_M$ and $MS_{Dox}-HA_H$ in water (pH 7.4). Data are presented as mean ± standard deviation (n=3).

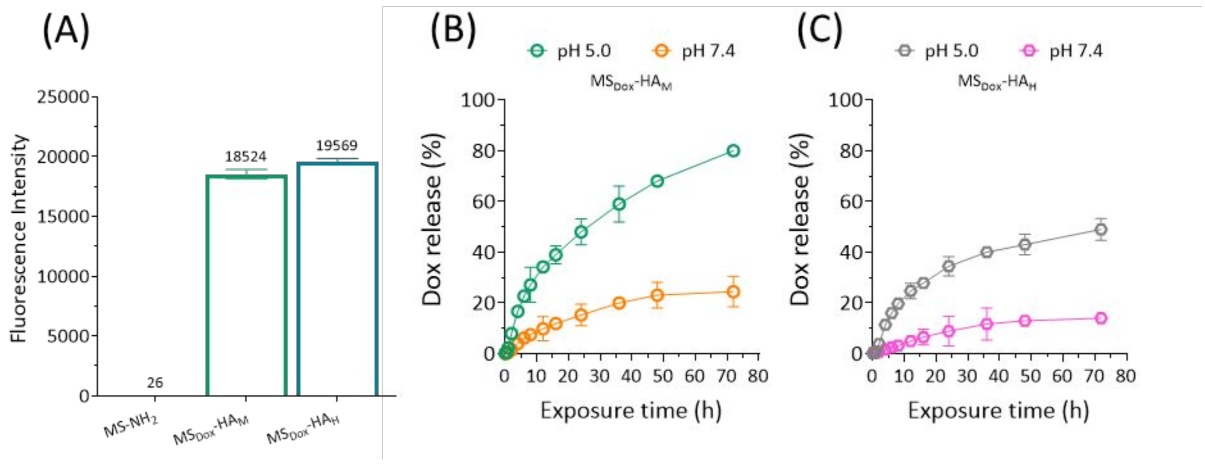

Figure S8 (A) Fluorescence intensity of MS_{Dox}-HA_M and MS_{Dox}-HA_H in water (pH 7.4). (B-C) Dox release profile (%) of MS_{Dox}-HA_M and MS_{Dox}-HA_H in pH 7.4 and 5.0 buffers. All data are presented as mean ± standard deviation (n=3).

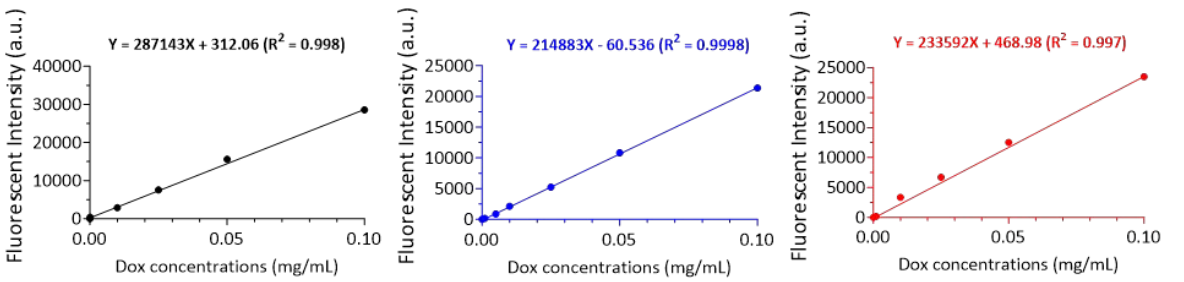

Figure S9 Standard curves (fluorescence) of Dox in water (pH 7.4), cacodylate buffer (pH 7.4), and cacodylate buffer (pH 5.0), respectively. All data are presented as mean ± standard deviation (n=3).

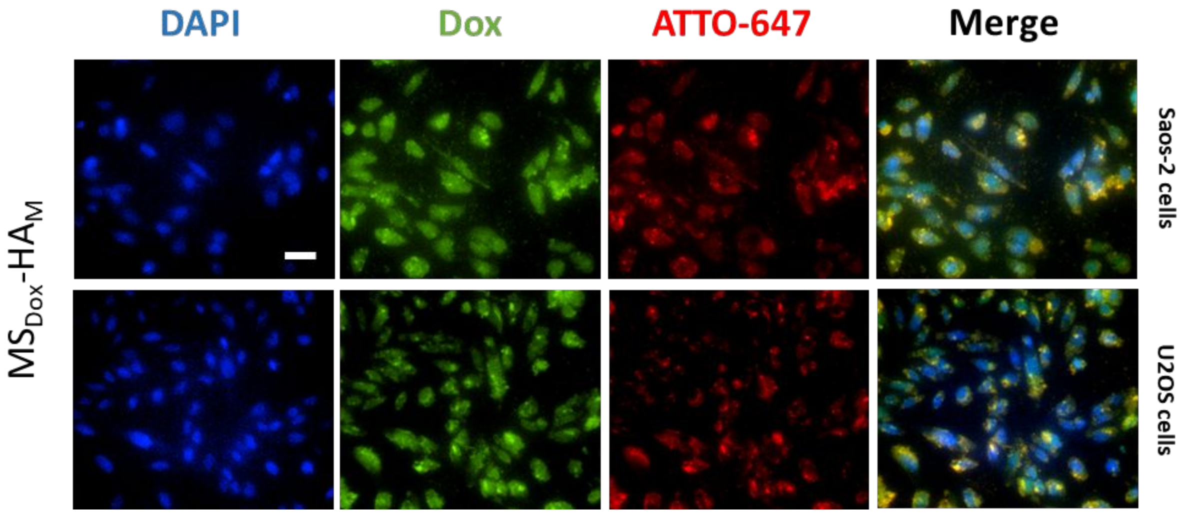

Figure S10 Intracellular uptake of MS_{Dox}-HA_M (100 µg/mL) in Saos-2 and U2OS cells after 12 h incubation; scale bar is 50 µm. Nuclei were stained with DAPI (blue channel). The green channel indicates loaded Dox. The red channel indicates the ATTO-647 dye labeling in MS_{Dox}-HA.

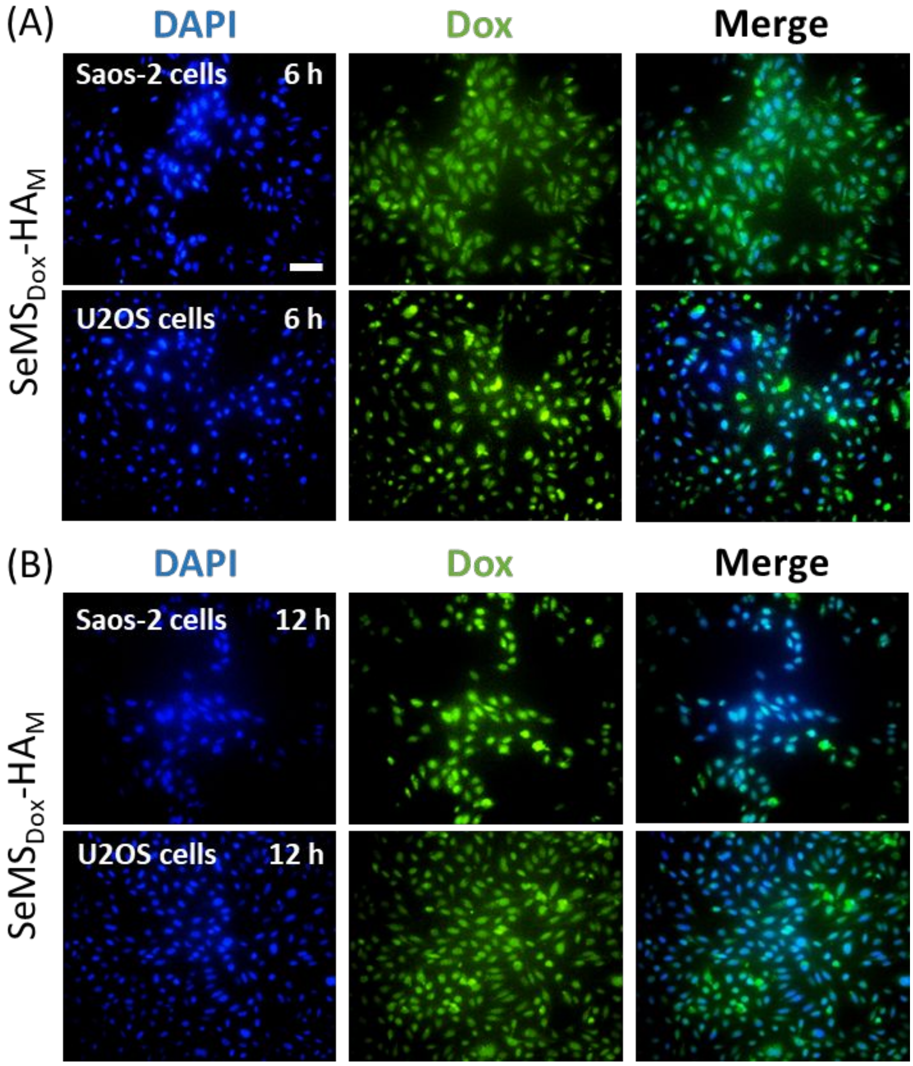

Figure S11 Cellular internalization of SeMS$_{Dox}$-HA$_M$ in OS cells after (A) 6 and (B) 12 h incubation; scale bar is 100 μm. The nanoparticle concentration was 100 μg/mL. Green channel indicates Dox loaded in SeMS$_{Dox}$-HA$_M$ to track the nanoparticle location in OS cells. Blue channel indicates cell nuclei to localize OS cells.

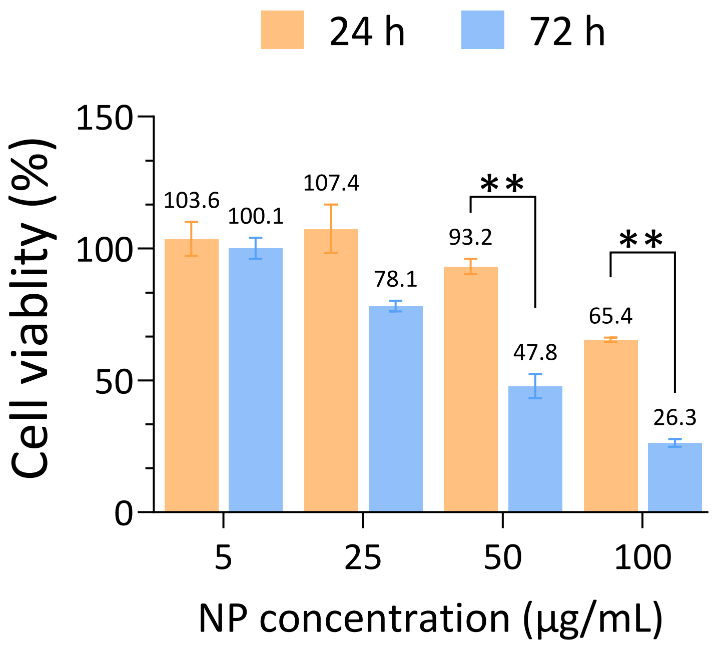

Figure S12 Cell viability of hMSCs after exposure to $SeMS_{Dox}$-HA_M for 24 and 72 h. All data are presented as mean ± standard deviation (n=3). * presents statistical significance (*: $p < 0.005$; **: $p < 0.0001$).

3. References

(1) Kauffman, M. K.; Kauffman, M. E.; Zhu, H.; Jia, Z.; Li, Y. R. Fluorescence-based assays for measuring doxorubicin in biological systems. *React. Oxyg. Species (Apex)* **2016**, *2* (6), 432.
